# Intranasal immunization with an RBD-hemagglutinin fusion protein harnesses preexisting immunity to enhance antigen-specific responses

**DOI:** 10.1172/JCI166827

**Published:** 2023-12-01

**Authors:** Atsushi Kawai, Nagisa Tokunoh, Eigo Kawahara, Shigeyuki Tamiya, Shinya Okamura, Chikako Ono, Jessica Anindita, Hiroki Tanaka, Hidetaka Akita, Sho Yamasaki, Jun Kunisawa, Toru Okamoto, Yoshiharu Matsuura, Toshiro Hirai, Yasuo Yoshioka

**Affiliations:** 1 Laboratory of Nano-Design for Innovative Drug Development, Graduate School of Pharmaceutical Sciences, and; 2 Vaccine Creation Group, BIKEN Innovative Vaccine Research Alliance Laboratories, Research Institute for Microbial Diseases, Osaka University, Osaka, Japan; 3 The Research Foundation for Microbial Diseases of Osaka University, Osaka, Japan.; 4 Department of Microbiology and Immunology, School of Pharmaceutical Sciences, Wakayama Medical University, Wakayama, Japan.; 5 Center for Infectious Disease Education and Research and; 6 Laboratory of Virus Control, Research Institute for Microbial Diseases, Osaka University, Osaka, Japan.; 7 Laboratory of DDS Design and Drug Disposition, Graduate School of Pharmaceutical Sciences, Chiba University, Chiba, Japan.; 8 Center for Advanced Modalities and DDS, Osaka University, Osaka, Japan.; 9 Laboratory of DDS Design and Drug Disposition, Graduate School of Pharmaceutical Sciences, Tohoku University, Miyagi, Japan.; 10 Laboratory of Molecular Immunology, Immunology Frontier Research Center, and; 11 Department of Molecular Immunology, Research Institute for Microbial Diseases, Osaka University, Osaka, Japan.; 12 Laboratory of Vaccine Materials and Laboratory of Gut Environmental System, Microbial Research Center for Health and Medicine, National Institutes of Biomedical Innovation, Health and Nutrition (NIBIOHN), Osaka, Japan.; 13 Institute for Advanced Co-Creation Studies, Research Institute for Microbial Diseases,; 14 Vaccine Creation Group, BIKEN Innovative Vaccine Research Alliance Laboratories, Institute for Open and Transdisciplinary Research Initiatives, and; 15 Global Center for Medical Engineering and Informatics, Osaka University, Osaka, Japan.

**Keywords:** Vaccines, Antigen, Antigen-presenting cells, Influenza

## Abstract

Intranasal vaccines are anticipated to be powerful tools for combating many infectious diseases, including SARS-CoV-2, because they induce not only systemic immunity but also mucosal immunity at the site of initial infection. However, they are generally inefficient in inducing an antigen-specific immune response without adjuvants. Here, we developed an adjuvant-free intranasal vaccine platform that utilizes the preexisting immunity induced by previous infection or vaccination to enhance vaccine effectiveness. We made RBD-HA, a fusion of the receptor-binding domain (RBD) of spike derived from SARS-CoV-2 as a vaccine target with HA derived from influenza A virus (IAV) as a carrier protein. Intranasal immunization of previously IAV-infected mice with RBD-HA without an adjuvant elicited robust production of RBD-specific systemic IgG and mucosal IgA by utilizing both HA-specific preexisting IgG and CD4^+^ T cells. Consequently, the mice were efficiently protected from SARS-CoV-2 infection. Additionally, we demonstrated the high versatility of this intranasal vaccine platform by assessing various vaccine antigens and preexisting immunity associated with a variety of infectious diseases. The results of this study suggest the promising potential of this intranasal vaccine platform to address problems associated with intranasal vaccines.

## Graphical abstract

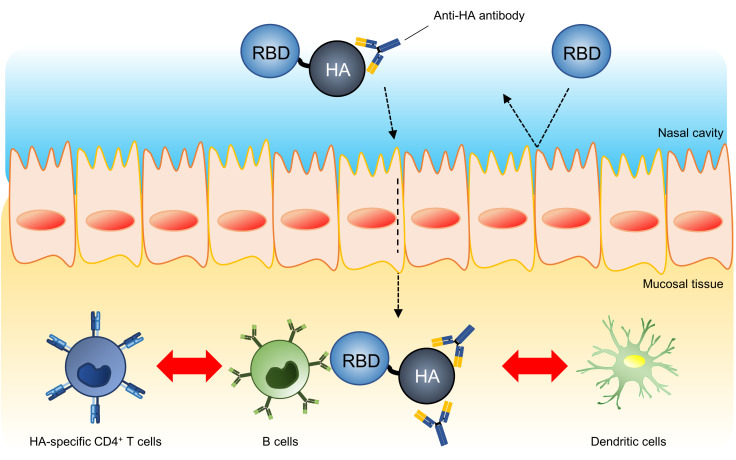

## Introduction

The SARS-CoV-2 pandemic has accelerated vaccine development at an unprecedented rate. Several types of COVID-19 vaccines, including mRNA and adenovirus-vector vaccines expressing the SARS-CoV-2 spike glycoprotein, provide highly effective protection and have been widely used (1, 2). Currently, approved COVID-19 vaccines are administered intramuscularly, inducing robust systemic immune responses, such as circulating antibodies and CD4^+^ and CD8^+^ T cells, and have demonstrated the ability to protect against severe disease and reduce mortality (1–3). Many pathogens, including the SARS-CoV-2 and influenza virus, initiate infections in the upper respiratory tract. However, traditional parenteral vaccines elicit poor mucosal immunity, as evidenced by secretory IgA in the upper respiratory tract (4, 5). Thus, they do not completely prevent viral infections or their transmission (6, 7). Hence, the development of intranasal vaccines capable of inducing IgA on mucosal surfaces as well as IgG in the systemic circulation is desired.

Subunit vaccines, which use pathogen-derived proteins or peptides as vaccine antigens, have several advantages over other vaccine types, such as live-attenuated vaccines and inactivated vaccines (8). These advantages include superior safety, easy upscaling of production, low production costs, and easy storage requirements. However, due to the mucosal barrier that blocks the delivery of antigens to antigen-presenting cells (APCs), such as DCs, macrophages, and B cells, intranasal subunit vaccines are inefficient in inducing an antigen-specific immune response. As a result, while attempts have been made to develop intranasal subunit vaccines used in combination with adjuvants, there are concerns about adverse reactions (9). For example, a human clinical trial of an intranasal influenza vaccine containing an inactivated influenza virus plus an adjuvant was discontinued due to suspicions that the combined use of adjuvant would cause Bell’s palsy in vaccinated individuals in rare cases (10). Therefore, no intranasal subunit vaccine has been approved. Given the problems, an adjuvant-free intranasal subunit vaccine with enhanced antigen delivery would be ideal.

The delivery of antigens to APCs is a key strategy for an effective vaccine (11). In intranasal vaccines, there are two major challenges to delivering antigens to APCs. The first is the mucosal epithelial barrier, which keeps antigens outside the body. Furthermore, even if antigens penetrate the first barrier, they need to be efficiently delivered to APCs and activated to trigger strong antigen-specific immune responses. However, these hurdles do not hold true for antigens against which we already have specific antibodies. Recently, several studies indicated that antibodies, such as IgA, in the nasal cavity aid the passage of bound antigens through the mucosal barrier (12–14). Furthermore, the interaction of the Fc portion of IgG with its receptors (FcγRs) on APCs substantially promotes antigen uptake and activation of APCs (15, 16). Therefore, utilizing preexisting immunity induced by previous infections may be an extremely effective tool for enhancing vaccine effectiveness. For example, influenza virus infection induces not only systemic IgG but also mucosal IgA and IgG to the HA glycoprotein on the influenza virus surface, and many human adults naturally have preexisting antibodies to HA from previous exposure to the seasonal influenza virus (17). Therefore, our study focused on the idea that a vaccine antigen fused with a carrier protein that recognizes preexisting antibodies could facilitate passage through the mucosal barrier and be picked up by APCs simultaneously.

Here, we made RBD-HA, a fusion of the receptor-binding domain (RBD) of the spike protein derived from SARS-CoV-2 as a vaccine target with HA, to test whether HA-specific preexisting immunity could be utilized. We showed that intranasal immunization of previously influenza virus–infected mice with RBD-HA without an adjuvant elicited a robust production of RBD-specific systemic IgG and mucosal IgA by utilizing HA-specific preexisting IgG and CD4^+^ T cells. Furthermore, RBD-HA protected mice in both the upper and lower respiratory tracts against SARS-CoV-2 infection. In addition, preexisting immunity induced by *Streptococcus pneumoniae* infection and an injectable mRNA vaccine can be utilized, suggesting high versatility of the vaccine system. Thus, we propose an adjuvant-free intranasal vaccine platform that could induce strong systemic and mucosal protective immunity.

## Results

### Intranasal vaccine with unadjuvanted RBD-HA induces antigen-specific systemic IgG and mucosal IgA.

Many studies have used large volumes (i.e., 20 to 30 μL/mouse) of vaccine-containing solutions as nasal vaccines in mouse models to immunize the upper respiratory and lower respiratory tracts, including the lungs; however, this approach might not accurately reﬂect nasal vaccine outcomes in humans. Previous studies have revealed that intranasal administration of a fluid volume less than 10 μL can limit antigen delivery to the upper respiratory tract of mice (18). Similarly, we found that mice treated intranasally with 7 μL of Evans blue dye showed no apparent staining in the airways and lungs, whereas distinct blue staining was confirmed following administration of 30 μL of treatment (Supplemental Figure 1A; supplemental material available online with this article; https://doi.org/10.1172/JCI166827DS1). Furthermore, we could not detect any luciferase activity in the bronchoalveolar lavage fluid (BALF) from mice that intranasally received 7 μL of recombinant luciferase protein, whereas significant luciferase activity was detected following administration of 30 μL of recombinant luciferase protein (Supplemental Figure 1B). Therefore, throughout the study, we used 7 μL of vaccine antigens as a model of nasal vaccine to immunize only the upper respiratory tract of each mouse.

**Figure 1:**
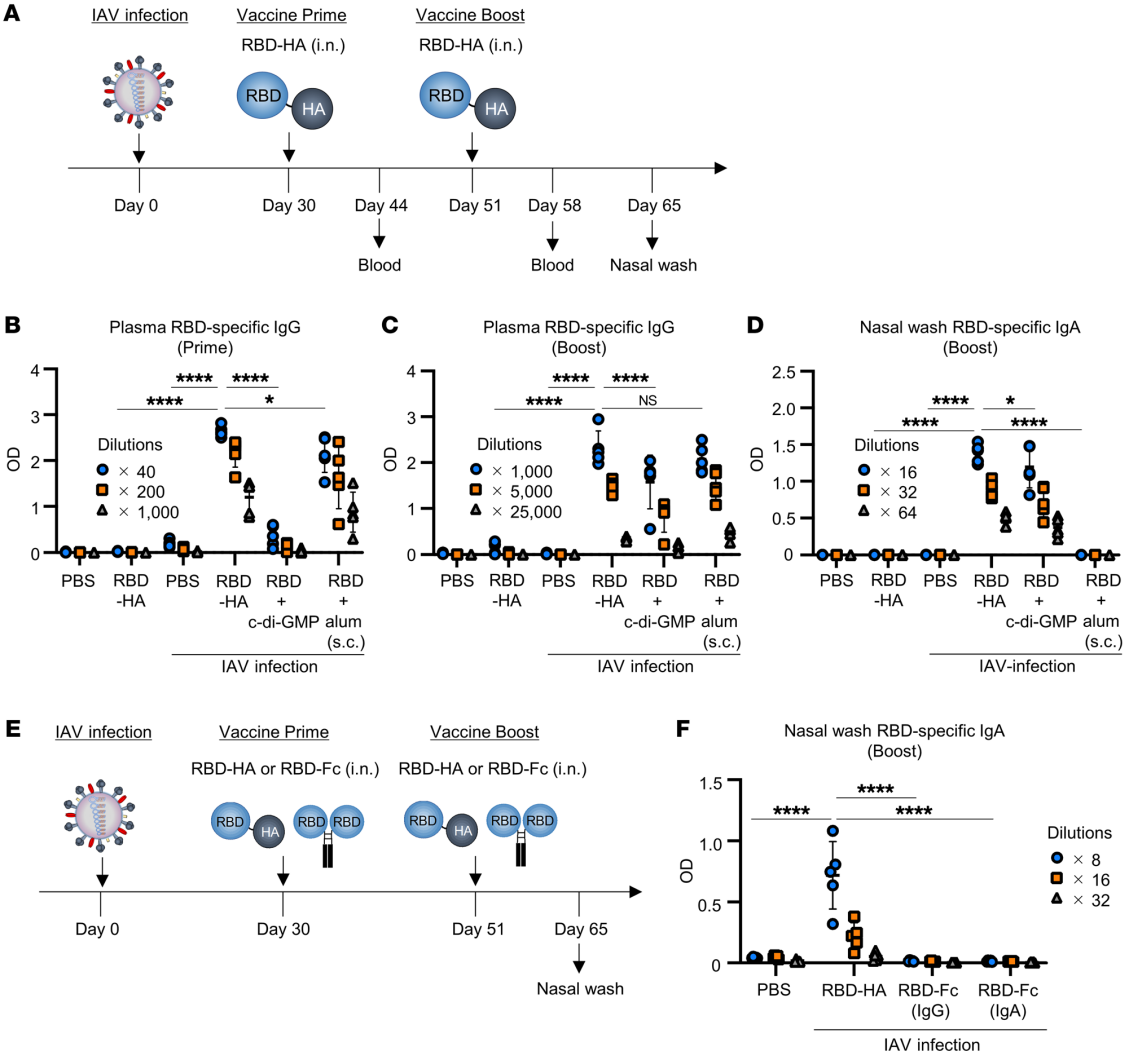
Adjuvant-free intranasal vaccination with RBD-HA induces both systemic IgG and mucosal IgA. (**A**) Experimental schematic: BALB/c mice were intranasally infected with IAV (IAV-mice), followed by intranasal immunization with RBD-HA without adjuvant, RBD plus c-di-GMP, and subcutaneous immunization with RBD plus alum at 30 and 51 days after IAV infection. (**B**–**D**) RBD-specific (**B**) plasma IgG levels after primary immunization, (**C**) plasma IgG levels after booster immunization, and (**D**) nasal wash IgA levels were evaluated using an ELISA after booster immunization. (**E**) Experimental schematic: IAV-mice were immunized intranasally with RBD-HA, RBD-Fc (IgG), or RBD-Fc (IgA) without an adjuvant at 30 and 51 days after IAV infection. (**F**) The RBD-specific IgA levels in nasal washes were evaluated using ELISA after booster immunization. Data are represented as means ± SD. (**A**–**F**) *n* = 5. Each experiment was performed more than twice. *P < 0.05; ****P < 0.0001, Tukey’s multiple-comparisons test.

To assess whether preexisting immunity could be utilized as vaccine antigen delivery carriers, we fused RBD as the target antigen with HA, which would be recognized by preexisting immunity. RBD-HA was generated in mammalian cells and purified using immobilized metal ions and size chromatography (Supplemental Figure 2, A and B). See complete unedited blots in the supplemental material. First, we determined whether RBD-HA induced antibody responses in the absence of adjuvants. BALB/c mice were infected with influenza A virus (IAV) in the upper respiratory tract (IAV-mice) to mimic a person who had experience of IAV infection. At 30 and 51 days after IAV infection, naive or IAV-mice were immunized intranasally with RBD-HA without an adjuvant (Figure 1A). As a positive control group, IAV-mice were immunized with RBD plus cyclic di-GMP (c-di-GMP), which has been previously used as an intranasal vaccine adjuvant in animal experiments (19, 20). For comparison with injectable vaccines, we immunized IAV-mice subcutaneously with RBD plus alum (scRBD-alum). We found that RBD-specific IgG in plasma was induced in IAV-mice immunized intranasally with RBD-HA at 14 days after primary immunization, but was not induced in naive mice that received intranasal RBD-HA (Figure 1B). Furthermore, RBD-specific IgG levels in plasma were significantly higher in IAV-mice immunized intranasally with RBD-HA than in IAV-mice treated with PBS, RBD plus c-di-GMP, or scRBD-alum at 14 days after primary immunization (Figure 1B). Seven days after the booster immunization, RBD-specific IgG levels were significantly higher in IAV-mice immunized with RBD-HA than in IAV-mice immunized with RBD plus c-di-GMP (Figure 1C). RBD-specific IgG levels in IAV-mice immunized with RBD-HA were comparable to those of scRBD-alum (Figure 1C). RBD-specific IgA levels in the nasal wash were significantly higher in IAV-mice immunized with RBD-HA than in IAV-mice immunized with RBD plus c-di-GMP at 14 days after booster immunization (Figure 1D). Conversely, IAV-mice immunized with RBD alone did not elicit RBD-specific IgA production in the nasal wash (Supplemental Figure 2C), indicating that our vaccine efficacy does not rely on a breakdown of mucosal immune tolerance by IAV infection. Similarly to the BALB/c mice results, RBD-specific IgA in the nasal wash was significantly higher in C57BL/6 IAV-mice immunized with RBD-HA than in the other groups 14 days after booster immunization (Supplemental Figure 2D). These results demonstrated that RBD-HA induces a robust mucosal and systemic antibody response in IAV-mice, even in the absence of the adjuvant, and regardless of mouse species. Additionally, we evaluated RBD-specific IgA in nasal wash and BALF after intranasal vaccination with RBD-HA using volumes of 7 μL and 30 μL. RBD-specific IgA levels in the nasal wash were significantly higher in IAV-mice immunized intranasally with 7 μL of RBD-HA than in those immunized intranasally with 30 μL of RBD-HA and subcutaneously with RBD-HA (Supplemental Figure 3A). Conversely, RBD-specific IgA levels in BALF were significantly higher in mice immunized intranasally with 30 μL of RBD-HA than in those immunized intranasally with 7 μL of RBD-HA and subcutaneously with RBD-HA (Supplemental Figure 3B). A recent study has shown that local immune responses are most strongly induced when antigens are present at the site (21). Together, these results suggest that, in terms of immune responses, our immunization is limited to the nasal cavity.

Several studies demonstrate that the fusion of vaccine antigens with the antibody Fc domain can enhance the immune response (22–24). Thus, to compare RBD-HA and Fc fusion antigens, IAV-mice were immunized with RBD-HA, RBD-Fc (IgG), or RBD-Fc (IgA) without an adjuvant (Figure 1E). We found that RBD-specific IgA levels in nasal wash were significantly higher in IAV-mice intranasally immunized with RBD-HA than in those immunized with RBD-Fc (IgG) and RBD-Fc (IgA) 14 days after booster immunization (Figure 1F). These results suggest this intranasal vaccine platform is superior to the traditional Fc fusion strategy.

Next, to measure antigen-specific T cell responses, we fused a spike containing both CD4^+^ and CD8^+^ T cell epitopes of SARS-CoV-2 as the target antigen with HA. Spike-specific IgG in plasma and IgA in nasal wash were significantly higher in IAV-mice immunized with spike-HA than in those immunized with trimeric spike plus c-di-GMP after booster immunization (Supplemental Figure 4, A and B). The neutralizing activity of antibodies induced by spike-HA immunization was evaluated using vesicular stomatitis virus–based (VSV-based) pseudotyped viruses displaying the Alpha spike of SARS-CoV-2. At all dilutions, nasal wash from IAV-mice immunized with spike-HA demonstrated superior neutralizing activity compared with that from IAV-mice immunized with trimeric spike plus c-di-GMP (Supplemental Figure 4C). This result suggested that antibodies induced by the spike-HA have robust neutralizing activity. Conversely, during ex vivo restimulation with spike, mice immunized with spike-HA did not induce IL-13 and IFN-γ production in either CD4^+^ or CD8^+^ T cells in the spleen, nasal-associated lymphoid tissue (NALT), or nasal passage compared with mice immunized with trimeric spike plus c-di-GMP (Supplemental Figure 5, A–C). In contrast to antibody induction, these results suggest that spike-HA is inefficient in inducing spike-specific T cell responses.

### Intranasal vaccination with RBD-HA protects against SARS-CoV-2 infection in both the upper and lower respiratory tracts.

**Figure 2:**
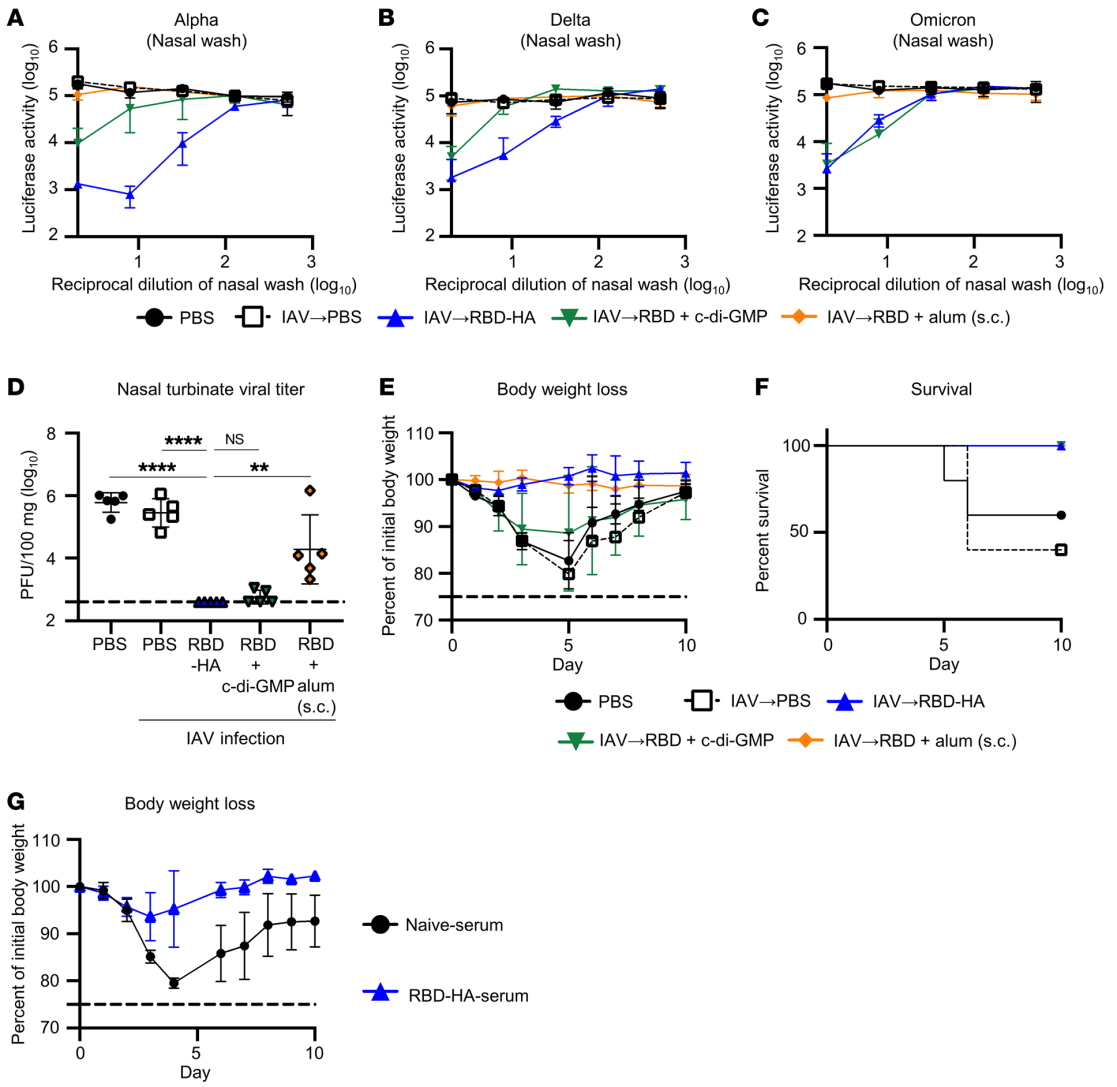
Intranasal RBD-HA vaccination protects mice from SARS-CoV-2 challenge in the upper and lower respiratory tracts. IAV-mice were intranasally immunized with RBD-HA or RBD plus c-di-GMP or were subcutaneously immunized with RBD plus alum at 30 and 51 days after IAV infection. (**A**–**C**) Measurement of neutralization against VSV-based pseudotyped viruses displaying (**A**) Alpha, (**B**) Delta, or (**C**) Omicron spike of SARS-CoV-2 in nasal wash. (**D**) Mice were challenged with SARS-CoV-2 to achieve upper respiratory tract infection. Three days after challenge, virus titers were evaluated in nasal turbinate tissues by plaque assay. (**E** and **F**) Mice were challenged with SARS-CoV-2 to achieve lower respiratory tract infection. Following virus challenge, the percentage changes in (**E**) body weight and (**F**) survival were monitored. (**G**) Serum collected from naive mice or IAV-mice immunized with RBD-HA (naive serum or RBD-HA serum, respectively) was transferred to naive mice. Mice were challenged with SARS-CoV-2 to achieve lower respiratory tract infection 24 hours after serum transfer. Following the virus challenge, the percentage changes in body weight were monitored. (**A**–**D** and **G**) *n* = 5; (**E** and **F**) *n* = 4–5. (**A**–**G**) Data are represented as means ± SD. (**D**) The dotted line represents the limit of detection. Each experiment was performed more than twice. (**D**) **P < 0.01; ****P < 0.0001, Tukey’s multiple-comparisons test.

To evaluate the neutralizing activity of antibodies induced by RBD-HA immunization, we used pseudotyped viruses displaying Alpha, Delta, or Omicron spike of SARS-CoV-2. Nasal washes obtained from IAV-mice immunized with RBD-HA neutralized Alpha and all other pseudotyped viruses (Figure 2, A–C). Even at the lowest dilutions, the nasal wash derived from scRBD-alum did not neutralize any of the pseudotyped viruses, mirroring the levels of IgA response against RBD (Figure 2, A–C).

In addition, we evaluated whether the immune responses induced by RBD-HA had a protective effect against SARS-CoV-2 infection. We used 2 SARS-CoV-2 virus challenge models in which the virus was administered intranasally to mice to infect either the upper respiratory tract (5 μL to the nares) or lower respiratory tract (20 μL to the nares). First, to assess whether there was a potential protective effect from prior IAV infection against SARS-CoV-2 infection, mice were infected with SARS-CoV-2 at different time points (4 days, 2 weeks, or 4 weeks) following IAV infection. As a result of lower respiratory tract infection, we observed that all IAV-infected mice showed a similar degree of body weight loss compared with that of previously IAV-uninfected mice, indicating that IAV infection does not provide protection against SARS-CoV-2 infection (Supplemental Figure 6). Virus titers of the IAV-mice immunized with RBD-HA after the upper respiratory tract infection were significantly lower than in the IAV-mice immunized with PBS and scRBD-alum, which were comparable to those of RBD plus c-di-GMP (Figure 2D). As a result of lower respiratory tract infection, body weight of PBS-treated naive-mice and the IAV-mice immunized with PBS or RBD plus c-di-GMP decreased after challenge (Figure 2E). In particular, the survival rates of PBS-treated naive mice and PBS-treated IAV-mice were approximately 60% and 40%, respectively (Figure 2F). All mice administered with RBD-HA and scRBD-alum were completely protected from body weight loss or death due to lower respiratory tract infection (Figure 2, E and F). To further evaluate the potential of antibodies induced by RBD-HA for protection against SARS-CoV-2 infection, serum collected from IAV-mice immunized with RBD-HA (RBD-HA-serum) was transferred to naive mice that were then challenged with lower respiratory tract infection. Although the body weight of mice with serum collected from naive mice (naive serum) decreased after the SARS-CoV-2 challenge, that of mice with transferred RBD-HA-serum remained unaltered (Figure 2G). These results suggest that the intranasal RBD-HA vaccine provides sufficient protection by inducing antibodies despite the absence of adjuvants.

### Antibodies induced by RBD-HA are maintained for a long period of time.

**Figure 3:**
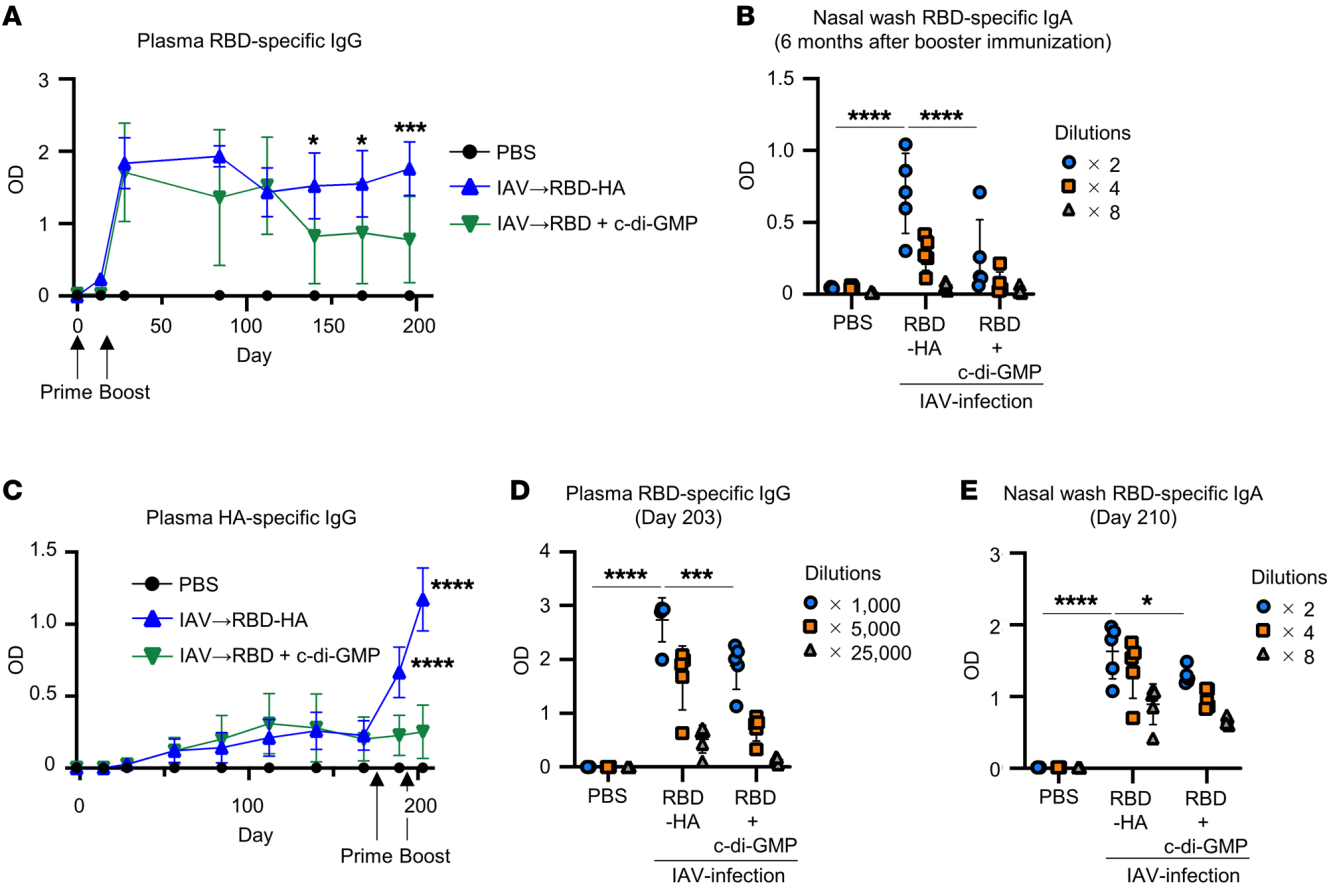
Antibodies elicited by RBD-HA exhibit long-term persistence. (**A**) Time-course changes of RBD-specific plasma IgG in IAV-mice immunized intranasally with RBD-HA or RBD plus c-di-GMP were evaluated using ELISA. Blood was collected at 0, 14, 28, 84, 112, 140, 168, and 196 days after primary immunization. We used 1,000-fold dilutions of plasma samples. (**B**) The RBD-specific IgA levels in nasal washes were evaluated using ELISA 203 days after primary immunization. (**C**) Time-course changes of HA-specific plasma IgG from IAV infection to intranasal immunization were evaluated using ELISA. Blood was collected at 0, 14, 28, 56, 84, 112, 140, 168, 189, and 203 days after IAV infection. We used 25,000-fold dilutions of plasma samples. (**D** and **E**) RBD-specific levels were evaluated using ELISA (**D**) plasma IgG levels and (**E**) nasal wash IgA levels after booster immunization. Data are represented as means ± SD. *n* = 5. Each experiment was performed more than twice. (**B**, **D**, and **E**) *P < 0.05; ***P < 0.001; ****P < 0.0001, Tukey’s multiple-comparisons test. (**A** and **C**) *P < 0.05; ***P < 0.001; ****P < 0.0001 between RBD-HA immunized IAV-mice and RBD plus c-di-GMP–immunized IAV-mice as indicated by Tukey’s multiple-comparisons test.

Next, we evaluated the durability of antibodies elicited by RBD-HA. RBD-specific IgG levels in plasma were maintained for approximately 6 months in IAV-mice immunized intranasally with RBD-HA after booster immunizations (Figure 3A). However, RBD-specific IgG levels in plasma were significantly lower in IAV-mice immunized with RBD plus c-di-GMP than in IAV-mice immunized with RBD-HA 4 months after booster immunizations (Figure 3A). Additionally, 6 months after the booster immunization, RBD-specific IgA levels in nasal washes were significantly higher in IAV-mice immunized with RBD-HA than in IAV-mice immunized with RBD plus c-di-GMP (Figure 3B). These results suggest that our vaccine strategy could induce durable antibodies.

Next, we evaluated whether RBD-HA could induce RBD-specific mucosal IgA and systemic IgG in mice long after IAV infection. IAV-mice were immunized intranasally with RBD-HA and RBD plus c-di-GMP on 175 and 196 days after IAV infection. The results showed that HA-specific IgG in IAV-mice increased until 112 or 140 days after infection compared with naive mice and subsequently reached equilibrium (Figure 3C). There was a significant increase in HA-specific IgG in RBD-HA–immunized IAV-mice compared with RBD plus c-di-GMP immunized IAV-mice 14 days after priming as well as 7 days after booster immunization (Figure 3C). In addition, we showed that IAV-mice administered with intranasal RBD-HA had significantly higher RBD-specific IgG in plasma and IgA in nasal wash levels than IAV-mice administered with RBD plus c-di-GMP (Figure 3, D and E). Therefore, these results suggested that RBD-HA is capable of inducing robust mucosal IgA and systemic IgG responses in mice, even during the extended period following IAV infection.

Humans are often exposed to many different pulmonary viral and bacterial infections, resulting in substantially different preexisting immunity compared with our IAV-mice, which were infected once with IAV prior to immunization. Therefore, we used *Mycoplasma pneumoniae* (Mp) and respiratory syncytial virus (RSV), both of which many people have a history of infection with, to evaluate whether a history of different pulmonary infections in IAV-mice affects the induction of immune responses by RBD-HA. Mice sequentially infected with Mp and RSV (Mp-RSV mice) were then infected with IAV. We confirmed that Mp-specific and fusion (F) protein–specific IgG in plasma, with F protein being the major membrane protein of RSV, was significantly higher in Mp-RSV mice than in control mice 28 days after IAV infection (Supplemental Figure 7, A and B). HA-specific IgG levels in plasma were not significantly different between Mp-RSV mice and control mice at 28 days after IAV infection (Supplemental Figure 7C). At days 30 and 51 after IAV infection, Mp-RSV and control mice were immunized intranasally with RBD-HA without an adjuvant. RBD-specific IgG in plasma and IgA in nasal washes induced by RBD-HA were not significantly different between Mp-RSV mice and control mice (Supplemental Figure 7, D and E). These results suggest that a history of different pulmonary infections has little impact on the immune response induced by RBD-HA in IAV-mice.

### HA-specific preexisting IgG in blood contributes to the immune responses induced by intranasal RBD-HA immunization.

**Figure 4:**
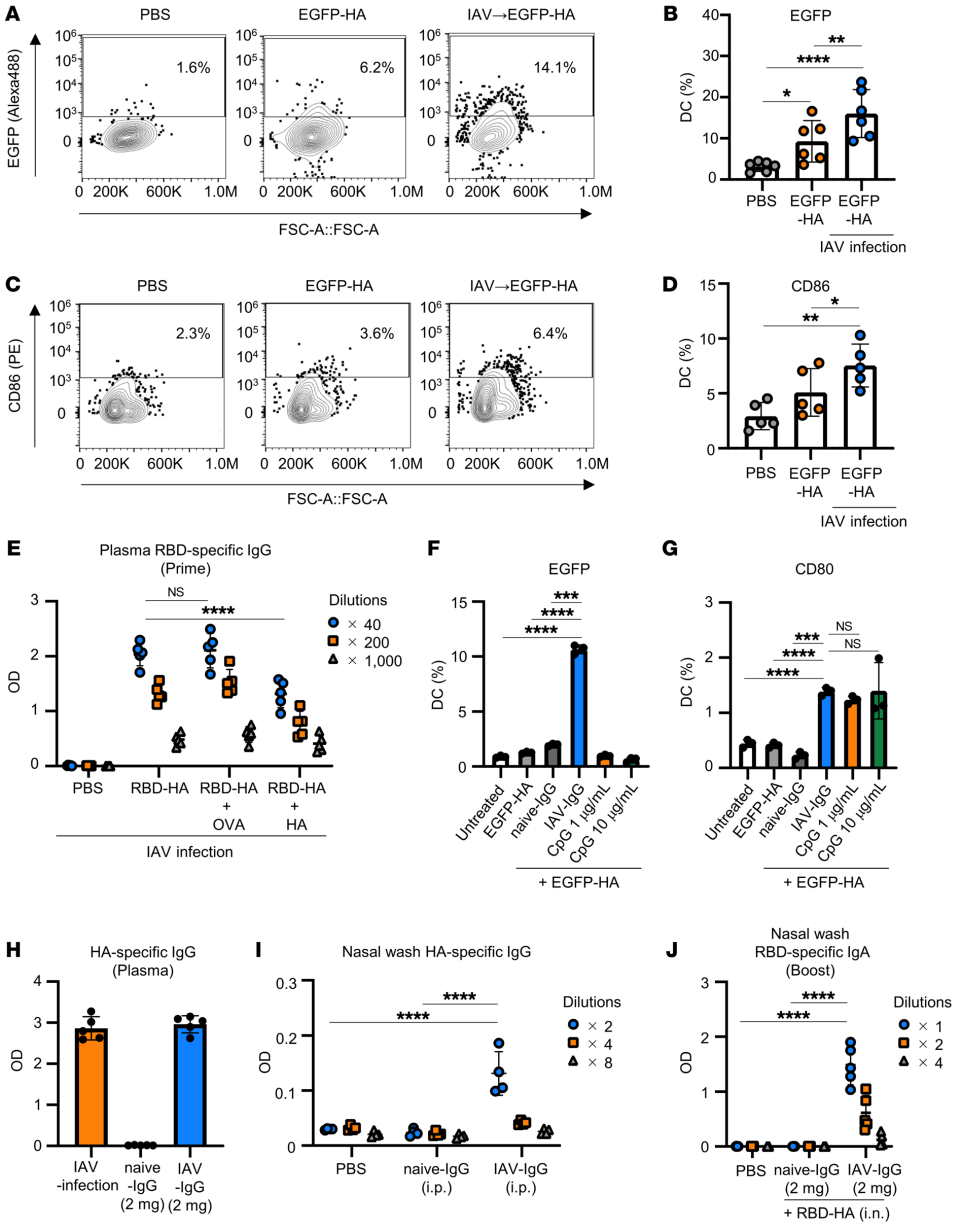
HA-specific preexisting IgG in blood contributes to the immune responses induced by intranasal vaccination with RBD-HA. (**A**–**D**) Naive or IAV-mice were immunized intranasally with EGFP-HA. Uptake of EGFP by DCs and costimulatory molecule expression on DCs were evaluated by flow cytometry. (**A** and **B**) Percentages of DCs positive for EGFP in nasal passage. (**C** and **D**) Percentages of DCs positive for CD86. (**E**) IAV-mice were intranasally immunized with RBD-HA plus OVA or HA. Levels of RBD-specific IgG were evaluated using ELISA. (**F** and **G**) Antigen uptake and costimulatory molecule expression in BMDCs were evaluated in vitro by flow cytometry. BMDCs were treated with EGFP-HA, EGFP-HA plus naive-IgG, IAV-IgG, or CpG ODN. (**F**) The percentages of BMDCs positive for EGFP. (**G**) The percentages of BMDCs positive for CD80. (**H**–**J**) BALB/c mice received 2 mg of purified naive-IgG or IAV-IgG and were immunized intranasally after 24 hours with RBD-HA. (**H** and **I**) Levels of HA-specific (**H**) plasma IgG and (**I**) nasal wash IgG were evaluated using ELISA 24 hours after passive transfer. (**J**) Levels of RBD-specific IgA in nasal wash after booster immunization were evaluated using ELISA. (**A** and **B**) *n* = 6; (**F** and **G**) *n* = 3; (**C**, **D**, **E**, **H**, and **J**) *n* = 5; (**I**) *n* = 4. (**B**, **D**–**J**) Data are represented as means ± SD. Each experiment was performed more than twice. *P < 0.05; **P < 0.01; ***P < 0.001; ****P < 0.0001, Tukey’s multiple-comparisons test.

To observe antigen uptake by DCs, IAV-mice were intranasally administered with EGFP-HA. Using flow cytometry, we analyzed DCs in the nasal passage and NALT as the main mucosal immune inductive site (Supplemental Figure 8, A and B). Six hours after immunization with EGFP-HA, more EGFP signal was detected in DCs from the nasal passage of IAV-mice than in naive mice (Figure 4, A and B). Furthermore, 24 hours after immunization, EGFP-HA induced enhanced expression of the activation marker CD86, a costimulatory molecule, on DCs in the NALT of IAV-mice compared with naive mice immunized with EGFP-HA (Figure 4, C and D). These results suggest that preexisting immunity promotes the uptake of antigens and further activates DCs.

We hypothesized that enhanced uptake of antigens by DCs was mediated to HA-specific preexisting antibodies. Therefore, to examine the importance of preexisting antibodies to HA in the immune response induced by RBD-HA, IAV-mice were immunized with RBD-HA plus HA to compete with preexisting HA-specific antibodies. As a control group, IAV-mice were immunized with RBD-HA plus OVA. RBD-specific IgG was significantly lower in IAV-mice immunized with RBD-HA plus HA versus RBD-HA or RBD-HA plus OVA (Figure 4E). Furthermore, to directly assess the contribution of IgG, it was purified from serum obtained from IAV-mice or naive mice (IAV-IgG or naive-IgG, respectively). We observed that IAV-IgG had the potential to promote uptake of antigens and enhance expression of the activation marker CD80, a costimulatory molecule, on DCs in vitro (Supplemental Figure 8C and Figure 4, F and G). To further elucidate the contribution of HA-specific preexisting IgG, 2 mg purified IAV-IgG was injected intraperitoneally into naive mice 24 hours prior to immunization with RBD-HA to mimic the levels of HA-specific IgG in blood induced by IAV infection (Figure 4H). Additionally, we found that HA-specific IgG was detected in nasal washes 24 hours after injection, indicating that circulating IAV-IgG in blood had access to the nasal cavity (Figure 4I). Mice pretreated with IAV-IgG induced a significantly higher RBD-specific IgG in plasma after both primary and booster immunization with RBD-HA compared with mice pretreated with naive-IgG (Supplemental Figure 9, A and B). Additionally, mice pretreated with 2 mg IAV-IgG induced significantly higher RBD-specific IgA in the nasal wash after booster immunization with RBD-HA than mice pretreated with naive-IgG (Figure 4J). These data indicate that HA-specific preexisting IgG can sufficiently promote RBD-specific mucosal IgA and systemic IgG production induced by intranasal RBD-HA vaccination.

### HA-specific IgG in the nasal cavity contributes to the immune response by intranasal RBD-HA immunization.

**Figure 5:**
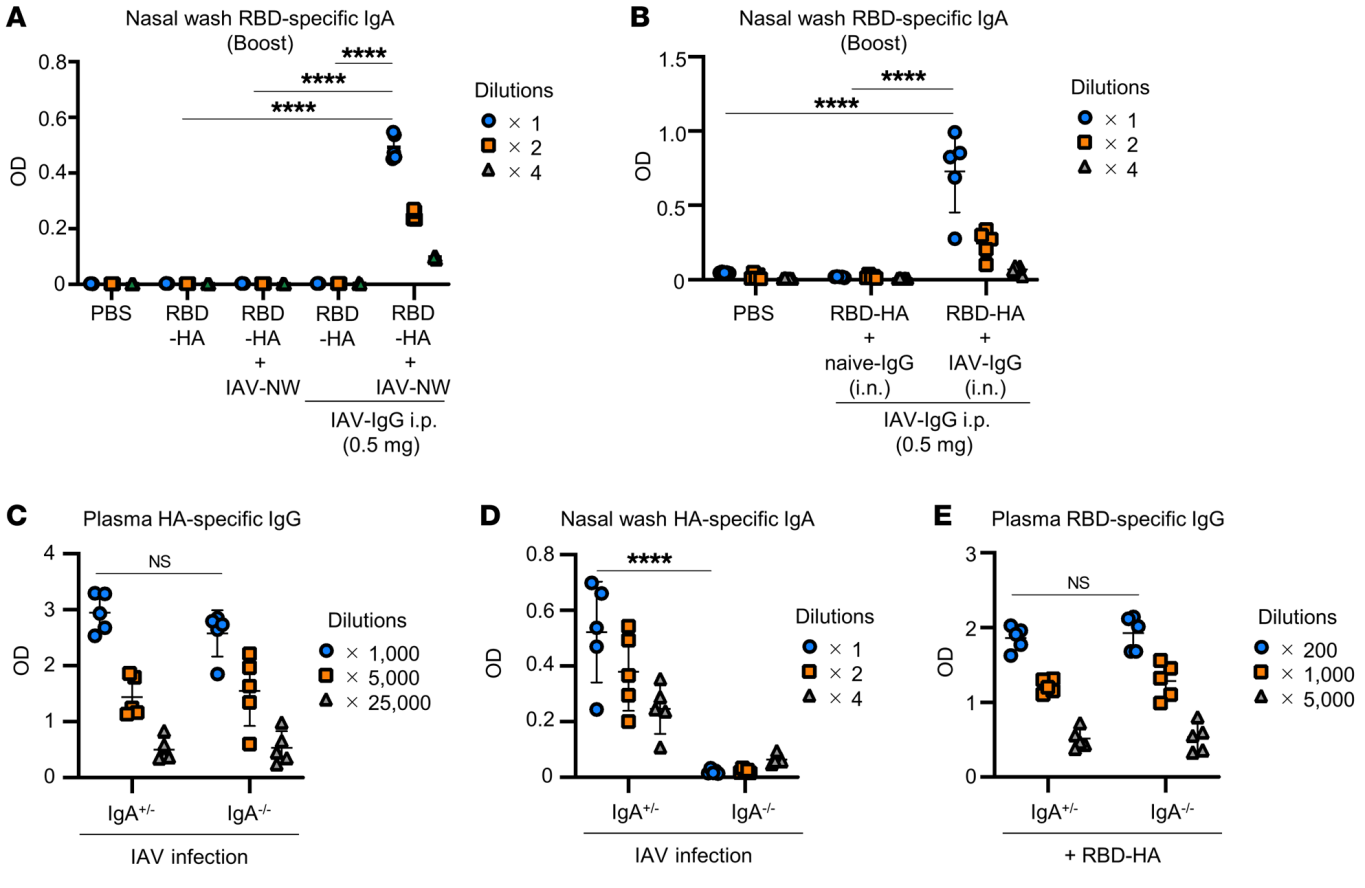
HA-specific preexisting IgG in nasal cavity contributes to the immune responses induced by intranasal vaccination with RBD-HA. (**A**) BALB/c mice received purified naive-IgG or IAV-IgG and were immunized intranasally after 24 hours with RBD-HA or RBD-HA plus IAV-NW. The levels of RBD-specific nasal wash IgA after booster immunizations were evaluated using ELISA. (**B**) BALB/c mice received purified IAV-IgG and were immunized intranasally after 24 hours with RBD-HA plus IAV-IgG. The levels of RBD-specific nasal wash IgA after booster immunization with RBD-HA were evaluated using ELISA. (**C**–**E**) IgA^+/–^ and IgA^–/–^ mice were intranasally infected with IAV, followed by intranasal immunization with RBD-HA at 30 and 51 days after IAV infection. (**C** and **D**) Levels of HA-specific (**C**) plasma IgG and (**D**) nasal wash IgA were evaluated using ELISA 28 days after IAV infection. (**E**) Levels of RBD-specific IgG after booster immunization with RBD-HA were evaluated using ELISA. (**A**–**E**) *n* = 5. (**A**–**E**) Data are represented as means ± SD. Each experiment was performed more than twice. ****P < 0.0001, Tukey’s multiple-comparisons test.

To assess the roles of HA-specific preexisting antibodies, such as IgG and IgA, in the nasal cavity, intranasal coadministration of RBD-HA and nasal washes obtained from IAV-mice (IAV-NW) was performed on naive mice. RBD-specific IgA was not detected in the mice that received a mixture of RBD-HA and IAV-NW (Figure 5A). In contrast, we found that IAV-NW enhanced RBD-specific IgA in nasal washes of mice pretreated intraperitoneally with 0.5 mg IAV-IgG; however, the amount of IAV-IgG alone was not enough to enhance RBD-specific IgA in the nasal cavity (Figure 5A). These results indicated that HA-specific antibodies in the nasal cavity could enhance induction of immune responses by intranasal RBD-HA in the presence of HA-specific IgG in blood. Furthermore, to evaluate the involvement of IgG in the nasal cavity, intranasal coadministration of RBD-HA and IAV-IgG was performed on mice pretreated intraperitoneally with 0.5 mg IAV-IgG. We observed that IAV-IgG significantly enhanced the production of RBD-specific IgA in the nasal wash of mice coadministered with RBD-HA and IAV-IgG compared with that of mice coadministrated with RBD-HA and naive-IgG (Figure 5B). These results suggested that the presence of IAV-IgG in both circulation and the nasal cavity synergistically promotes the induction of RBD-specific antibodies in our intranasal RBD-HA vaccine system.

In addition, we used IgA-knockout mice (IgA^–/–^) to evaluate the involvement of IgA present in the nasal cavity. We confirmed that HA-specific IgA was not detected in IgA^–/–^ mice at 28 days after IAV infection, while HA-specific IgG in plasma was not significantly different from that of control IgA heterozygous mice (IgA^+/–^) and IgA^–/–^ mice (Figure 5, C and D). At 30 and 51 days after IAV infection, IAV-infected IgA^+/–^ and IgA^–/–^ mice were immunized intranasally with RBD-HA. The RBD-specific IgG levels in plasma induced by RBD-HA were not significantly different between IAV-infected IgA^+/–^ and IgA^–/–^ mice at 7 days after booster immunization with RBD-HA (Figure 5E). Considering these results, it is likely that the enhanced immune response resulting from the mixed administration of IAV-NW and RBD-HA was mediated by IgG rather than IgA present in the nasal wash.

### HA-specific preexisting CD4^+^ T cells contribute to the immune responses induced by RBD-HA intranasal immunization.

**Figure 6:**
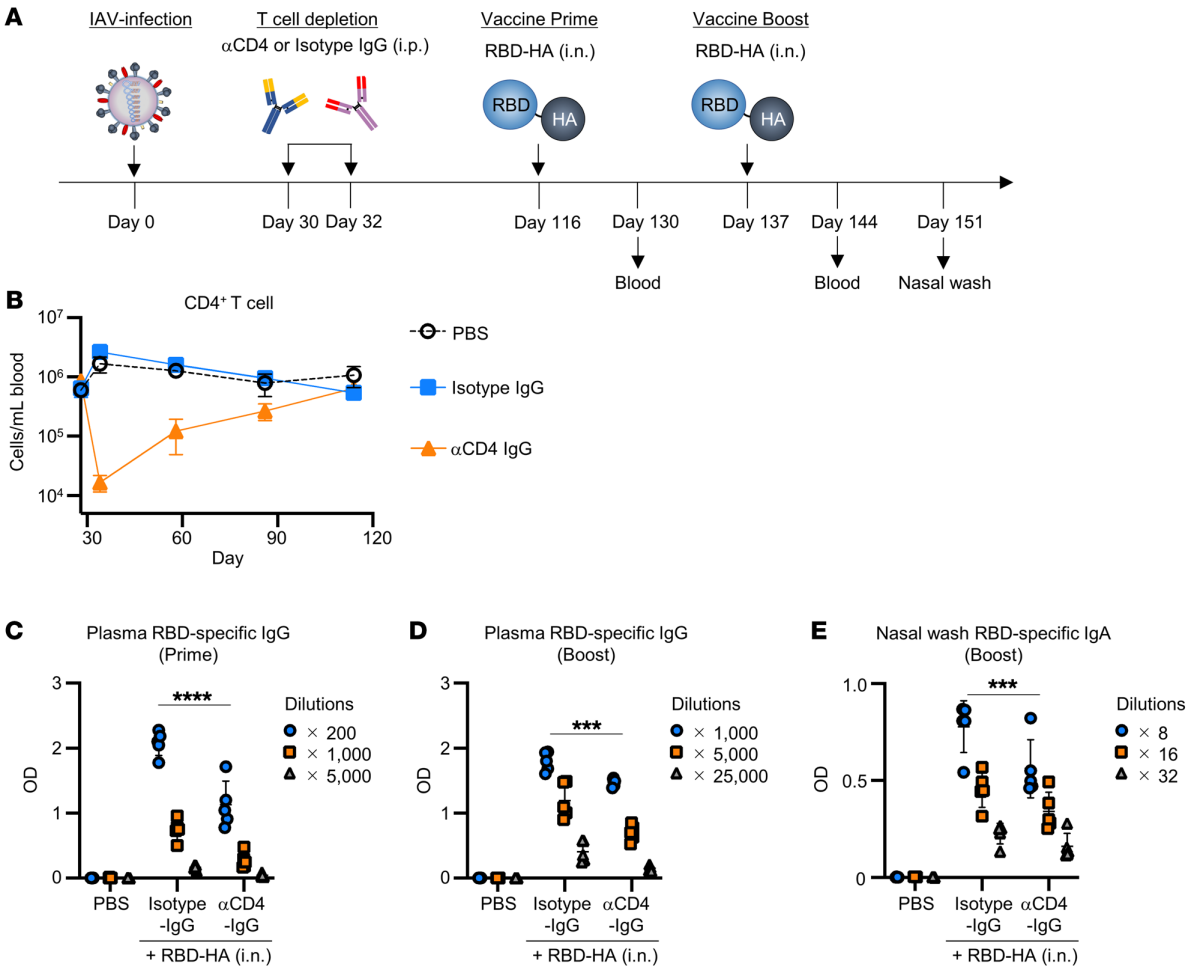
Preexisting HA-specific CD4^+^ T cells contribute to the immune responses induced by intranasal vaccination with RBD-HA. (**A**) Experimental schematic: for depletion of HA-specific preexisting CD4^+^ T cells, IAV-mice were intraperitoneally injected with 200 μg anti-CD4 antibody (GK1.5) or isotype antibody at 30 and 32 days, respectively, after IAV infection. (**B**) CD4^+^ T cell numbers in blood were monitored from 28 to 114 days after infection. Blood was collected at 28, 34, 58, 86, and 114 days after IAV infection. (**C**–**E**) IAV-mice were immunized intranasally with RBD-HA at 116 and 137 days after IAV infection. The RBD-specific levels were evaluated using ELISA (**C**) plasma IgG levels after prime, (**D**) plasma IgG levels after boost, and (**E**) nasal wash IgA levels after booster immunization. (**B**–**E**) Data are represented as means ± SD. *n* = 5. Each experiment was performed more than twice. ***P < 0.001; ****P < 0.0001, Tukey’s multiple-comparisons test.

IAV infection induced not only HA-specific antibodies,but also led to the production of HA-specific CD4^+^ T cells. Thus, to confirm the importance of HA-specific memory CD4^+^ T cells in the induction of immune responses by RBD-HA, IAV-mice were given CD4^+^ T cell–depleting antibody (αCD4-IgG) 30 and 32 days after IAV infection (Figure 6A). We found that CD4^+^ T cells were depleted from the peripheral blood after αCD4-IgG injection (Supplemental Figure 10A). Mice recovered naive CD4^+^ T cells at 114 days as much as naive mice (Figure 6B). This recovery rate is consistent with a previous report (25). IAV-mice treated with αCD4-IgG showed no difference in the amount of HA-specific IgG in the blood compared with IAV-mice treated with isotype-IgG at 114 days after infection (Supplemental Figure 10B). At 116 and 137 days after IAV infection, mice were intranasally immunized with RBD-HA. We observed that the RBD-specific IgG in plasma and IgA in nasal wash levels in IAV-mice treated with αCD4-IgG were significantly lower than those in IAV-mice treated with isotype-IgG (Figure 6, C–E). These results suggest that HA-specific memory CD4^+^ T cells contribute to enhancement of the immune response in IAV-mice intranasally immunized with RBD-HA.

### The intranasal vaccine platform is extremely versatile.

**Figure 7:**
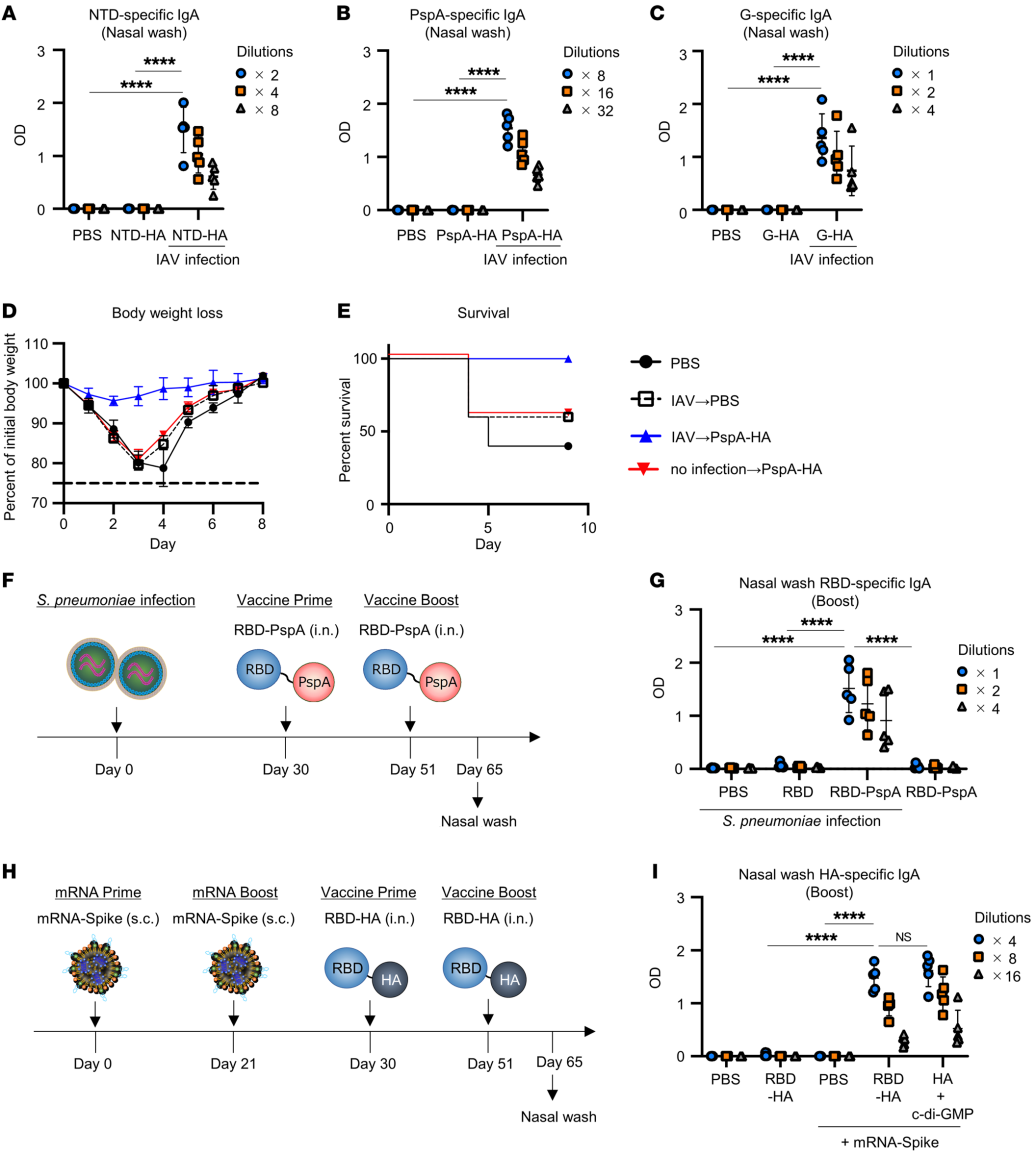
Intranasal subunit vaccine platform that utilizes preexisting immunity is highly versatile. (**A**–**E**) IAV-mice were immunized intranasally with (**A**) NTD-HA, (**B**, **D**, and **E**) PspA-HA, or (**C**) G-HA at 30 and 51 days after IAV infection. (**A**–**C**) Levels of (**A**) NTD-, (**B**) PspA-, and (**C**) G-specific IgA in nasal wash were evaluated using ELISA. (**D** and **E**) Fourteen days after booster immunization, IAV-mice were challenged with *S*. *pneumoniae* to achieve lower respiratory tract infection. The percentage changes in (**D**) body weight and (**E**) survival were monitored after challenge with *S*. *pneumoniae*. (**F**) Experimental schematic: BALB/c mice were infected intranasally with *S*. *pneumoniae*, followed by intranasal immunization with RBD-PspA or RBD without adjuvant at 30 and 51 days after *S*. *pneumoniae* infection. (**G**) The RBD-specific nasal wash IgA levels were evaluated using ELISA. (**H**) Experimental schematic: BALB/c mice were subcutaneously immunized with 1 μg of mRNA vaccine encoding SARS-CoV-2 spike twice, followed by intranasal immunization with RBD-HA or HA plus c-di-GMP at 30 and 51 days after mRNA vaccine. (**I**) The HA-specific nasal wash IgA levels were evaluated using ELISA. (**A**–**I**) Data are represented as means ± SD. *n* = 5. Each experiment was performed more than twice. ****P < 0.0001, Tukey’s multiple-comparisons test.

To confirm the versatility of this intranasal vaccine platform against various types of antigens, we fused HA with the N-terminal domain (NTD) of spike derived from SARS-CoV-2, pneumococcal surface protein A (PspA) derived from *S*. *pneumoniae*, or the conserved central domain from major surface G glycoprotein (G) derived from RSV. IAV-mice were immunized intranasally twice with NTD-HA, PspA-HA, or G-HA without adjuvant. We found that antigen-specific IgG in plasma and IgA in nasal wash were significantly higher in IAV-mice immunized with NTD-HA, PspA-HA, or G-HA than in the naive-mice (Figure 7, A–C, and Supplemental Figure 11, A–C). Furthermore, the IAV-mice were challenged with *S*. *pneumoniae* infection after the booster immunization. IAV-mice immunized with PspA-HA were completely protected from weight loss or death (Figure 7, D and E).

We further evaluated whether other preexisting immunity, as well as HA-specific immunity, could be utilized. To determine whether PspA-specific preexisting immunity induced by *S*. *pneumoniae* infection can be utilized to enhance immune responses, we fused an RBD as the target antigen with PspA. On 30 and 51 days after infection with *S*. *pneumoniae* in the upper respiratory tract, mice were immunized intranasally with RBD-PspA without an adjuvant (Figure 7F). After booster immunization, we found that *S*. *pneumoniae*–infected mice that received RBD-PspA intranasally induced significantly higher RBD-specific IgA levels in the nasal wash and IgG levels in plasma than other groups (Figure 7G and Supplemental Figure 11D). These results suggest that our intranasal vaccine platform is highly versatile.

With the appearance of SARS-CoV-2, many people have been vaccinated with mRNA and have spike-specific antibodies and CD4^+^ T cells. Thus, we determined whether preexisting immunity induced by an mRNA vaccine can be utilized. On 30 and 51 days after immunization with 1 μg of mRNA encoding spike of SARS-CoV-2 twice (mRNA-mice), mice were immunized intranasally with RBD-HA without adjuvant, PBS, or HA plus c-di-GMP (Figure 7H). We found that the mRNA vaccine induced significantly higher spike-specific IgG levels in plasma than naive mice 7 days after booster immunization with the mRNA vaccine (Supplemental Figure 12A). In addition, mRNA-mice that received intranasal RBD-HA induced significantly higher HA-specific IgA levels in the nasal wash in naive mice immunized with RBD-HA (Figure 7I). This was comparable to that of HA plus c-di-GMP (Figure 7I). Furthermore, to assess whether the intensity of preexisting immunity affects antibody production by RBD-HA, we immunized mice with RBD-HA after administering the mRNA vaccine at lower doses. We observed that spike-specific IgG levels in plasma were correlated with the amount of mRNA vaccine administered (Supplemental Figure 12B). Although there were no significant differences in HA-specific IgA induced by RBD-HA in mice pretreated with 1 or 0.3 μg of mRNA, mice pretreated with 0.1, 0.03, or 0.01 μg of mRNA showed a dose-dependent decrease in HA-specific IgA induced by RBD-HA (Supplemental Figure 12C). This result suggested that there was a correlation between vaccine efficacy of RBD-HA and preexisting immunity.

## Discussion

In this study, we showed that intranasal vaccination with RBD-HA induced robust mucosal and systemic immune responses in IAV-mice without the use of any adjuvants. Our results revealed that preexisting IgG is sufficient to induce RBD-specific antibodies by RBD-HA, suggesting that preexisting IgG is extremely important for our intranasal vaccine platform. We demonstrate that the intranasal coadministration of RBD-HA and IAV-IgG into mice pretreated intraperitoneally with 0.5 mg IAV-IgG enhanced the RBD-specific mucosal IgA. Additionally, our data revealed that some of the systemically treated IgG reached the nasal cavity. A recent study showed that circulating antibodies can reach the respiratory epithelium, but they cannot access the olfactory epithelium (26). These results indicate that the IgG in the respiratory epithelium, which can originate from circulating IgG in blood, also contributes to the induction of antibodies by intranasal RBD-HA vaccination. Moreover, we observed that the uptake of EGFP-HA was enhanced by mucosal DCs in the IAV-mice. A recent report indicated that albumin-binding antigens are capable of neonatal Fc receptor–mediated (FcRn-mediated) uptake across the mucosal barrier (27). FcRn is widely expressed on mucosal epithelial cells in adult animals and humans, and it plays a crucial role in the bidirectional transcytosis of both IgG and albumin, facilitating their recycling (28–30). Although the detailed mechanism needs to be further elucidated in future studies, based on previous reports and our data, it can be hypothesized that HA-specific IgG in the nasal cavity assists in HA-conjugated vaccine antigen passage through the mucosal barrier potentially via interaction with FcRn.

However, we found that intranasal coadministration of RBD-HA and IAV-NW could not induce an immune response in the absence of IAV-IgG in the blood. This result suggested that circulating IgG has other roles related to inducing immune responses in addition to supplying IgG to the nasal cavity. Our results showed that IAV-IgG promotes uptake of the antigen and activation in DCs in vitro. DCs are known to express several Fcγ receptors, such as FcγRI, FcγRIIb, FcγRIII, and FcγRIV (31–33). The FcγR-IgG interaction supposedly induces the internalization of antigen-IgG (immune complex) and activation of DCs, and improves antigen presentation on MHC class I and class II molecules (34–36). It has been previously shown that the administration of antigens, such as tetanus toxoid and hepatitis B antigen, in the form of an immune complex increases immunogenicity by 10- to 1,000-fold (15, 16). Additionally, IgG is also reported to be present in NALT as well as in the blood (37, 38). Thus, our results indicate that the antigen that passed through the mucosal epithelium formed immune complexes with IgG in NALT and the blood, which facilitated the uptake of antigen into the DCs and further activated them.

We demonstrated that IgA is not necessary for the induction of RBD-specific IgG in IAV-infected mice following RBD-HA intranasal vaccination. Conversely, several reports show that IgA can enhance the transcytosis of bound antigens across the mucosal epithelium through Dectin-1 and Siglec-5 (12–14). In our experiments, we evaluated the involvement of IgA under conditions where IgG was sufficiently present to induce immune responses by RBD-HA. It is possible that IgA may contribute to inducing immune responses when IgG levels are insufficient to do so; nevertheless, further investigation is required.

The present study confirms the importance of the contribution of HA-specific preexisting CD4^+^ T cells in enhancing the immune response through CD4^+^ T cell–depletion experiments. However, unexpected nonspecific effects, particularly the depletion of Tregs, might also occur. Several studies have established that helper CD4^+^ T cells primed to a carrier protein increase antibody responses to haptens fused with the carrier protein (39–41). Thus, our results indicate that HA fused to RBD may act as a carrier protein in IAV-mice and enhances antibody production against RBD. As IAV infection is shown to induce HA-specific systemic IgG, nasal cavity IgG, and CD4^+^ T cells (42–44), the strong immune response induced in IAV-mice immunized intranasally with RBD-HA without an adjuvant might be a comprehensive result of these contributions.

This study also used monomeric spike-HA. It is well known that monomeric spike generally exhibits lower immunogenicity compared with that of trimeric spike (45). Our results showed that antibodies induced by the spike-HA consisting of monomeric spike have robust neutralizing activity, suggesting that spike-HA has sufficient immunogenicity to induce an immune response. However, both spike-HA and RBD-HA used monomeric HA as a carrier protein. Therefore, the RBD-HA with trimeric HA might be a better option in terms of more effective protection against both SARS-CoV-2 and influenza virus for future studies.

Immune complexes strongly induce immune responses and are reported to be used in vaccine strategies. For example, mixing antigens with the antigen-specific antibodies and administering them in the form of immune complexes has been used for an efficient vaccine system for many years in animal experiment (15, 16). However, this strategy is complicated because of the need to mix the antigen and antibody prior to administration and has not yet been applied to intranasal vaccines. As another approach, several studies demonstrated that the fusion of protein antigens with antibody Fc domains can enhance intranasal vaccination (46, 47). This Fc fusion strategy enhanced antigen-specific systemic and mucosal antibody responses. To the best of our knowledge, the effectiveness of this approach, utilizing intranasal immunization with Fc fused to an antigen without an adjuvant, in inducing immune responses has not been evaluated. We tested and discovered that our intranasal vaccine platform induced stronger immune responses than Fc fusion antigens without use of any of the adjuvants. One substantial distinction between the Fc fusion strategy and our vaccine platform is that this vaccine platform can utilize polyclonal antibodies. In general, except for FcγRI, which can engage monomeric IgG with high affinity, FcγRs exhibit low affinity for IgG and can only interact with multimeric IgG immune complexes or opsonized cells generated during an infectious challenge (48). Therefore, our findings that our vaccine platform was superior to the Fc fusion strategy suggest that this is due to the availability of polyclonal antibodies in addition to the availability of both antibodies and CD4^+^ T cells.

Recently, a vaccine strategy was reported to induce mucosal immunity by intranasal boosters with spike proteins after primary vaccination with parental mRNA expressing SARS-CoV-2 spike (49). This vaccine-boosting strategy, called “prime and spike,” resulted in robust resident memory B cell and T cell responses and mucosal IgA. It is similar to our vaccine strategy that utilizes preexisting immunity, but ours induces immunity to the vaccine antigen, which is different from the antigen that the preexisting immunity specifically recognizes. Furthermore, we identified the critical roles of antigen-specific IgG and CD4^+^ T cells in preexisting immunity to induce strong immunity to the vaccine antigen in our strategy, which, we believe, will promote our understanding in this field.

Given the characteristics of our intranasal vaccine platform, the choice of utilizing preexisting immunity is critical. Our results indicated IgG levels in the blood could be a predictive marker for the efficacy of our vaccine strategy. For example, most adults have had tetanus and diphtheria vaccinations, and clinical reports show that approximately 97% of the population has serum antibodies to tetanus and diphtheria (50). Moreover, a recent serologic study found that most of the subjects (77%) had a moderate seropositive rate (HAI ≥1:40) for IAV, indicating that antibodies against IAV exist in the majority of the population (17). Therefore, the utilization and selection of these preexisting antibodies is extremely useful in our intranasal vaccine platform and may affect its efficacy. Furthermore, our intranasal vaccine platform may not necessarily rely on vaccination or infection history. Recent reports have demonstrated that natural antibodies against sugars of bacterial origin are strongly induced and well conserved in humans. For example, the anti-Gal antibody is the most abundant natural antibody in humans, constituting approximately 1% of serum IgG levels (51). While several vaccine strategies utilizing these natural antibodies in injectable vaccines have been reported (52, 53), there have been no reports of their application in intranasal vaccines. Thus, further investigations are needed to determine whether natural antibodies are available as carrier antibodies in intranasal vaccines to improve their robustness and versatility.

In humans, antibody titers to HA increase in the weeks following infection or vaccination and then decrease over time (54). Therefore, it is likely that there are differences in the antibody levels present in different people. We have shown that induction of HA-specific antibodies by RBD-HA correlated with doses of pretreated mRNA vaccines and their induced spike-specific IgG. This result suggested that the efficiency of our vaccine strategy might vary depending on the level of preexisting immunity. On the other hand, as shown in Figure 3C, immunization with RBD-HA increased not only antibodies to the newly introduced vaccine antigen (i.e., RBD) but also to the HA used as a carrier. This suggested that several doses of our vaccines could work in people who have less preexisting immunity to the carrier because antibodies to the carrier protein can be also boosted.

There are several limitations to our study. In experiments with using a mouse model, our results suggested that a history of previous infection does not affect the immune response induced by RBD-HA. However, our experiment was performed with mice in a clean environment, which does not accurately mimic the human environment where exposure to various pathogens often occurs. Future studies are needed to investigate whether our vaccine strategy is effective in humans who have been exposed to a wide variety of infections and live in diverse environments. Despite the limitation, this study provides important insights into the mechanisms with which this intranasal vaccine platform utilizes preexisting immunity to elicit immune responses.

## Methods

### Viruses and bacteria.

The H1N1 influenza virus strain A/California/7/2009 was provided by Hideki Asanuma (National Institute of Infectious Diseases, Tokyo, Japan). SARS-CoV-2 (MA10) strain was generated using the circular polymerase extension reaction (CPER)method as previously described (55). The SARS-CoV-2 NIID strain (2019-nCoV_Japan_TY_WK-5212020) serves as the backbone of this MA10. The MA10 contains 7 mutations, including nsp4: T295I, nsp7: K2R, nsp8: E23G, S: Q493K/Q498Y, P499T, and orf6: F7S, which have been reported as adaptive mutations introduced in SARS-CoV-2 during serial passages in BALB/c mice by Leist et al. (56). The FH strain of Mp was purchased from ATCC. RSV (strain A2) was provided by Takehiko Shibata (Department of Microbiology, Tokyo Medical University, Tokyo, Japan).

### Mice.

Male BALB/c and C57BL/6 mice (aged 6 to 7 weeks) were purchased from SLC. Male IgA-deficient (IgA^–/–^) BALB/c mice (aged 6 to 7 weeks) were obtained from Mutant Mouse Regional Resource Centers (MMRRC) (57). They were housed in a room with a 12-hour light/12-hour dark cycle and had unrestricted access to food and water.

### Plasmid construction.

The sequences for the NTD, RBD, and spike were derived from the SARS-CoV-2 spike sequence (Wuhan-1, GenBank MN908947.3). The HA sequence was derived from the IAV (A/California/7/2009, GenBank ACV82259.1). The F and G protein sequences were derived from RSV (A2, GenBank AAB59858.1 for the F protein and AAB59857.1 for the G protein). The PspA sequence was derived from *S*. *pneumoniae* (WU2, GenBank AF071814). The cDNAs of NTD, RBD, F, G, and HA were cloned into the pcDNA3.1 expression plasmid (Thermo Fisher Scientific). The cDNA of the ectodomain of F (amino acids 1-513) contains substitutions at P102A, S155C, S190F, V207L, S290C, I379V, and M447V. The cDNA of the ectodomain of spike (amino acids 1–1208) contains a glycine substitution at 614 (D614G), proline substitutions at 986 and 987 (K986P, V987P), and a GSAS substitution at the furin cleavage site (R682G, R683S, R685S). The foldon sequence (GYIPEAPRDGQAYVRKDGEWVLLSTFL) from the bacteriophage T4 fibritin was inserted at the C-terminus of F protein and spike to generate a trimeric protein. The cDNA of PspA with an N-terminal His tag was cloned into a pET28a vector (Merck Millipore). RBD fused to the N-terminus of the Fc region of IgG1 (GenBank AAK53870.1) or IgA (GenBank BAL37291.1) was cloned into pcDNA3.1. The detailed plasmid design is shown in Supplemental Figure 13.

### Protein purification.

All proteins were generated as previously described (58, 59). Detailed methodology is described in Supplemental Methods. For SDS-PAGE, purified proteins were mixed 1:1 (vol/vol) in sample buffer solution (Nacalai Tesque) and heated at 95°C for 5 minutes before being loaded onto a 10% Mini-PROTEAN TGX Precast Protein Gel (Bio-Rad). After electrophoresis, the gels were stained with Coomassie brilliant blue according to standard protocols.

To purify IgG, serum samples were passed through a protein G column (GE Healthcare) equilibrated with 20 mM phosphate buffer (pH 7.0) to allow binding of total IgG using an AKTA explorer chromatography system. After washing with phosphate buffer, the total IgG was eluted in 100 mM glycine HCl buffer (pH 2.7) and the eluted solution was immediately neutralized with 1M Tris-HCl buffer (pH 9.0). The buffer of the total IgG solution was then exchanged with PBS. The amount of protein was quantified using a Pierce BCA Protein Assay Kit (Thermo Fisher Scientific) with a bovine serum albumin standard.

### Vaccination.

For subsequent immunization after IAV infection, anesthetized naive mice were infected intranasally with 3.0 × 10^3^ TCID_50_ of IAV in a total volume of 5 μL of PBS (2.5 μL per nostril). On days 30 and 51 after infection, mice were immunized intranasally with RBD (10 μg/mouse), RBD-HA (10 μg/mouse), spike-HA (10 μg/mouse), NTD-HA (10 μg/mouse), PspA-HA (10 μg/mouse), G-HA (10 μg/mouse), EGFP-HA (32 μg/mouse), RBD-Fc (IgG) (10 μg/mouse), or RBD-Fc (IgA) (10 μg/mouse) in a total volume of 7 μL (3.5 μL per nostril) under anesthesia and immunized with RBD (10 μg/mouse) plus c-di-GMP (5 μg/mouse; InvivoGen), CpG ODN (CpG K3, 5 μg/mouse; Gene Design), or poly(I:C) (5 μg/mouse; InvivoGen) in a total volume of 7 μL (3.5 μL per nostril) under anesthesia. As shown in Supplemental Figure 3, on days 30 and 51 after infection, mice were immunized intranasally with RBD-HA (10 μg/mouse) with a total volume of 7 μL (3.5 μL per nostril) or 30 μL (15 μL per nostril) under anesthesia. As shown in Figure 3C, on days 175 and 196 after IAV infection, mice were immunized intranasally with RBD-HA (10 μg/mouse) or RBD (10 μg/mouse) plus c-di-GMP (5 μg/mouse) with a total volume of 7 μL (3.5 μL per nostril) under anesthesia. As shown in Figure 4E, on days 30 and 51 after IAV infection, mice were immunized intranasally with RBD-HA (1 μg/mouse) plus HA or OVA (50 μg/mouse) in a total volume of 7 μL (3.5 μL per nostril) under anesthesia.

For subcutaneous immunization, on days 30 and 51 after infection, mice were inoculated at the base of the tail with RBD (10 μg/mouse) plus Alhydrogel 2% (InvivoGen) as alum adjuvant (100 μg/mouse) in a total volume of 50 μL.

For subsequent immunization with *S*. *pneumoniae*, anesthetized naive mice were infected intranasally with 4 × 10^6^ CFU of *S*. *pneumoniae* (WU2) in a total volume of 5 μL of PBS (2.5 μL per nostril). On days 30 and 51 after infection, mice were immunized intranasally with RBD-PspA (10 μg/mouse) or RBD (10 μg/mouse) in a total volume of 7 μL (3.5 μL per nostril) under anesthesia.

For subsequent immunization after mRNA vaccination, naive mice were inoculated at the base of the tail on days 0 and 21, with 0.01, 0.03, 0.1, 0.3, or 1 μg of mRNA expressing SARS-CoV-2 spike. On days 30 and 51 after mRNA vaccination, mice were intranasally immunized with RBD-HA (10 μg/mouse) or HA (10 μg/mouse) plus c-di-GMP (5 μg/mouse) in a total volume of 7 μL (3.5 μL per nostril) under anesthesia.

### Detection of antigen-specific antibodies.

ELISA was used to detect antigen-specific IgG and IgA in plasma and nasal wash samples. ELISA plates were coated overnight at 4°C with antigen (1 μg/mL for plasma; 10 μg/mL for nasal wash) in carbonate buffer. The coated plates were then incubated with blocking solution (1% Block Ace; DS Pharma Biomedical) for 1 hour at room temperature. Plasma and nasal wash samples were serially diluted before being added to the antigen-coated plates. After incubation for 2 hours at room temperature, the sample-containing plates were incubated with HRP-conjugated goat anti-mouse IgG (catalog AP503; Merck Millipore) for 1 hour at room temperature or with biotin-conjugated goat anti-mouse IgA (catalog 1040-08; Southern Biotech) for 2 hours at room temperature, followed by the addition of HRP-conjugated streptavidin (Thermo Fisher Scientific). The color reaction was developed with tetramethyl benzidine (Nacalai Tesque), stopped with 2 N H_2_SO_4_, and measured at the OD_450_ to OD_570_ on a microplate reader (Power Wave HT, BioTek).

### Pseudovirus neutralization assay.

VeroE6/TMPRSS2 was seeded (1.2 × 10^4^ cells) in a volume of 100 μL on 96-well half-white plates (Greiner BIO-ONE) and incubated in DMEM high glucose with 2% (v/v) heat-inactivated FBS (Sigma-Aldrich), 1% (v/v) penicillin-streptomycin (Fujifilm) for 24 hours at 37°C. On the day of infection, nasal wash samples were heat inactivated for 30 minutes at 56°C. Nasal wash samples were tested using a starting undiluted solution with five 4-fold serial dilutions. Serial dilutions were mixed 1:1 with pseudotyped viruses, which is a replication-deficient VSV bearing the SARS-CoV-2 spike protein, and incubated for 1 hour at 37°C. Growth medium was then aspirated from the cells, replaced with 50 μL of nasal wash/virus mixture, and incubated at 37°C for 48 hours. Following 48 hours of infection, 50 μL of ONE-Glo-EX Reagent (Promega), which was equal to the volume of the culture medium, was pipetted into each well and mixed. Luminescence was measured using a microplate reader (Powerscan HT, DS Pharma Biomedical).

### The SARS-CoV-2 challenge.

Mouse-adapted SARS-CoV-2 was grown in VeroE6/TMPRSS2 cells, and their titer was measured using a plaque assay. Fourteen days after the booster immunization, immunized mice were challenged with SARS-CoV-2. For upper respiratory tract infections, anesthetized mice were challenged intranasally with 5 × 10^4^ PFU in 5 μL (2.5 μL per nostril). After 3 days, nasal turbinates were homogenized, and virus titers were evaluated by plaque assay. For lower respiratory tract infection, anesthetized mice were challenged intranasally with 2 × 10^5^ PFU in 20 μL (10 μL per nostril). The body weights and survival rates of the challenged mice were monitored for 10 days after challenge. The humane end point was set at 25% body weight loss relative to day 0. We defined the day on which the mice weighed less than 75% of their body weight on day 0 as the day of death.

### Serum passive transfer.

As described above, IAV-mice were immunized intranasally with RBD-HA twice. Naive-serum was obtained from the serum of naive mice. RBD-HA serum was obtained from mice immunized with RBD-HA 7 days after booster immunization. Serum obtained from individual mice was pooled. For serum-transfer experiments, mice were intraperitoneally administered 400 μL of naive serum or RBD-HA serum 24 hours before challenge with SARS-CoV-2.

### The S. pneumoniae challenge.

On the 14 days after the booster immunization, immunized mice were intranasally challenged with the WU2 strain of *S*. *pneumoniae*. Anesthetized mice were challenged intranasally with 5 *×* 10^6^ CFU of *S*. *pneumoniae* in 30 μL of PBS (15 μL per nostril). The body weights and survival rates of the challenged mice were monitored for 8 days after challenge.

### Flow cytometry.

To evaluate the DC response in the NALT and nasal passage, naive or IAV-mice were immunized intranasally with EGFP-HA. After 6 and 24 hours, mice were euthanized, and NALT and nasal passage lymphocytes were collected. Cells were analyzed using flow cytometry. For evaluating bone marrow–derived DC (BMDC) response in vitro, BMDCs (5 × 10^5^ cells) were incubated with EGFP-HA (1 μg/mL) plus naive-IgG (20 μg/mL), IAV-IgG (20 μg/mL), or CpG ODN (1 or 10 μg/mL) at 37°C in 96-well plates. After 6 and 24 hours, cells were analyzed using flow cytometry. Flow cytometric analysis was performed using an Attune NxT Flow Cytometer (Thermo Fisher Scientific). FlowJo software version 10.8.1 (TreeStar) was used for analysis. Detailed methodology is described in the Supplemental Methods.

### BMDC cultures and treatment.

1 × 10^7^ Bone marrow cells per well were cultured in tissue culture–treated 6-well plates in 4 mL of RPMI 1640 supplemented with 10% FBS, 1% penicillin-streptomycin, 50 mM 2-mercaptoethanol, and GM-CSF (20 ng/mL, PeproTech). After 48 hours, half of the medium was removed and replaced with new medium supplemented with GM-CSF (20 ng/mL) warmed at 37°C. At 72 hours, the culture medium was entirely discarded and replaced with fresh, warmed medium containing GM-CSF (20 ng/mL). On day 6, nonadherent cells in the culture supernatant and loosely adherent cells harvested by gentle washing with PBS were pooled and used as the starting material for most experiments.

### Adoptive transfer of purified IgG and nasal wash.

Naive-IgG was obtained from the serum of naive mice. IAV-IgG was obtained from the serum of mice that had been infected with IAV in the upper respiratory tract. Naive mice were intraperitoneally injected with 2 mg of naive- or IAV-IgG in 300 μL of PBS 24 hours before immunization with RBD-HA (10 μg/mouse).

To evaluate the contribution of antibodies in the nasal cavity, nasal washes were collected into 400 μL of PBS from IAV-mice (IAV-NW) 30 days after IAV infection. IAV-NW was concentrated approximately 20-fold by ultrafiltration (Merck Millipore) and used for adoptive transfer. Naive mice were immunized intranasally on days 0 and 21 with a mixture of RBD-HA (10 μg/mouse) and IAV-NW. To evaluate the contribution of IgG in the nasal cavity, we intranasally immunized mice pretreated intraperitoneally with 0.5 mg IAV-IgG with a mixture of RBD-HA (10 μg/mouse) and 50 μg of naive- or IAV-IgG.

### CD4^+^ T cell depletion.

To deplete CD4^+^ T cells, mice were injected intraperitoneally with 200 μg anti-CD4 antibody (clone GK1.5; Bio X Cell) or anti-keyhole limpet hemocyanin antibody as an isotype control (clone LTF-2; Bio X Cell) in 300 μL PBS at 30 and 32 days after IAV infection. Plasma samples were collected at days 34, 58, 86, and 114 after IAV infection to observe CD4^+^ T cell depletion and naive CD4^+^ T cell recovery. On days 116 and 137 after infection, mice were immunized intranasally with RBD-HA (10 μg/mouse) in a total volume of 7 μL (3.5 μL per nostril) under anesthesia.

### Statistics.

Statistical analyses were performed using Prism 9 software version 9.4.0 (GraphPad Software). All data are presented as means ± SD. Significant differences were determined using Tukey’s test. *P* < 0.05 was considered statistically significant.

### Study approval.

All animal experiments were performed in accordance with Osaka University’s Institutional Guidelines for the Ethical Treatment of Animals and were approved by the Animal Care and Use Committee of the Research Institute for Microbial Diseases, Osaka University (protocols BIKEN-AP-R01-15-2 and BIKEN-AP-R02-09-0). All experiments using viruses were approved by the Institutional Review Board of the Research Institute for Microbial Diseases, Osaka University (protocols BIKEN-00006-009, BIKEN-00137-045, BIKEN-00012-005, BIKEN-00138-002, and BIKEN-00224-001).

### Data availability.

All reagents used in this study will be made available upon reasonable request to the corresponding author. Values for all data points in graphs are reported in the Supporting Data Values file.

## Author contributions

AK, TH, and YY designed the study. AK, NT, EK, ST, SO, CO, JA, HT, HA, SY, JK, TO, and YM performed the experiments. AK, TH, and YY wrote the manuscript.

## Supplementary Material

Supplemental data

Supporting data values

## Version 1 12/01/2023

Electronic publication
