# Supplementary material for: Intranasal immunization with an RBD-hemagglutinin fusion protein harnesses preexisting immunity to enhance antigen-specific responses
Source: J Clin Invest. 2023 Dec 1;133(23):e166827. doi: 10.1172/JCI166827 (PMC10688985; doi:10.1172/JCI166827)
Supplement: Supplemental data [file jci-133-166827-s128.pdf]

Supplemental Methods

Recombinant proteins

The plasmid was transfected into 7.5 × 10^7 Expi293F cells in 30 mL medium using ExpiFectamine 293 Reagent. Cells were incubated at 37°C, 8% CO_2 on an orbital shaker (120 r/min) for 18 h before adding ExpiFectamine 293 Transfection enhancers 1 and 2 and the incubation of cells was continued. After incubation, the supernatant was collected. The pET28a PspA plasmid was transformed into *Escherichia coli* BL21 (DE3) cells (New England Biolabs, Beverly, MA, USA). PspA protein expression was induced by adding 0.5 mM isopropyl-β-D-1-thiogalactopyranoside (Nacalai Tesque, Kyoto, Japan), and then shaken for 4 h at 37°C. After incubation, the cells were lysed by sonication and the supernatant was collected. The soluble protein was purified using an AKTA explorer chromatography system equipped with a Ni-Sepharose HisTrap FF column (GE Healthcare, Chicago, IL, USA) and a Superose 6 Increase 10/300 GL column (GE Healthcare) for size-exclusion chromatography.

Anesthetic preparation

The anesthetic was prepared as a mixture of medetomidine (ZENOAQ, Koriyama, Japan), midazoram (Maruishi Pharmaceutical Co., Ltd, Osaka, Japan), and butorphanol (Meiji Seika Pharma Co., Ltd, Tokyo, Japan). Medetomidine (0.3 mg/kg b.w/mouse), midazoram (4.0 mg/kg b.w/mouse), and butorphanol (0.3 mg/kg b.w/mouse) were administrated intraperitoneally to mice in saline solution (Otsuka Pharmaceutical Co., Ltd, Tokyo, Japan).

Administration of Evans Blue dye and luciferase (Supplemental Figure 1)

Mice were administered intranasally with Evans Blue dye (Nacalai Tesque) at volumes of 7 μL and 30 μL. After 24 h, the airways and lungs were collected for observation. To evaluate luciferase activity, mice were administered intranasally with 1 × 10^6 units of luciferase protein (Sigma-Aldrich, St. Louis, MO, USA) at volumes of 7 μL and 30 μL or inoculated at the base of the tail at a total volume of 50 μL. Two h later, nasal wash and BALF were collected in 400 μL and 1 mL of PBS, respectively. Each sample was then mixed 1:1 with luciferin (Promega, Madison, WI, USA). Luminescence was measured using a microplate reader (Powerscan HT, DS Pharma Biomedical, Osaka, Japan).

Sequential infection (Supplemental Figure 7)

The mice were infected intranasally with 5 × 10^6 PFU of Mp in 5 μL (2.5 μL per nostril) and 1 × 10^5 PFU of RSV in 5 μL of PBS (2.5 μL per nostril) at seven days post-Mp infection. Seven days after the RSV infection (Mp-RSV mice), Mp-RSV mice and control mice were infected intranasally with 3 × 10^3 $TCID_{50}$ of IAV in a total volume of 5 μL of PBS (2.5 μL per nostril). On days 30 and 51 post-IAV infection, mice were immunized intranasally with RBD-HA (10 μg/mouse) at a total volume of 7 μL (3.5 μL per nostril) under anesthesia.

Supplemental Methods

Flow cytometry

Figure 4: To evaluate the DC response in the NALT and nasal passage, naive- or IAV-mice were immunized intranasally with EGFP-HA. After 6 and 24 h, the mice were euthanized, and NALT and nasal passage lymphocytes were collected. Cells were blocked with anti-mouse CD16/CD32 antibody (1:100 dilution; clone: 93; BioLegend, San Diego, CA, USA) and were stained with Fixable Viability Dye eFluor 780 (Thermo Fisher Scientific, Hampton, NH, USA), Alexa647 anti-mouse CD45 antibody (1:200 dilution; clone: 30-F11; BioLegend), PerCP/Cy5.5 anti-mouse CD11c antibody (1:200 dilution; clone: N418; BioLegend), BV421 anti-mouse I-A/I-E antibody (1:200 dilution; clone: M5/114.15.2; BioLegend), PE anti-mouse CD86 antibody (1:200 dilution; clone: GL-1; BioLegend) in PBS with 2% FBS, 1 mM EDTA (DOJINDO, Kumamoto, Japan), and 0.05% sodium azide (WAKO, Saitama, Japan) for 30 min at 4°C in dark, followed by intracellular Alexa488 anti-GFP antibody (1:200 dilution; clone: FM264G; BioLegend) staining using a BD Cytofix/Cytoperm™ Fixation/ Permeablization solution Kit (BD Biosciences, Sparks, MO, USA). For evaluating BMDCs response in vitro, BMDCs (5×10^5 cells) were incubated with EGFP-HA (1 μg/mL) plus naive-IgG (20 μg/mL), IAV-IgG (20 μg/mL), or CpG ODN (1 or 10 μg/mL) at 37°C in 96-well plates. 6 and 24 h later, cells were blocked with anti-mouse CD16/CD32 antibody and were stained with Fixable Viability Dye eFluor 780, PerCP/Cy5.5 anti-mouse CD11c antibody, APC anti-mouse CD80 antibody (1:200 dilution; clone: 16-10A1; BioLegend), BV421 anti-mouse I-A/I-E antibody, PE/Cy7 anti-mouse CD11b antibody (1:200 dilution; clone: M1/70; BioLegend) in PBS with 2% FBS, 1 mM EDTA, and 0.05% sodium azide for 30 min at 4°C in dark, followed by intracellular Alexa488 anti-GFP antibody staining using a BD Cytofix/Cytoperm™ Fixation/ Permeablization solution Kit.

Figure 6: To determine the number of $CD4^+$ T cells in the cells collected from blood, cells were blocked with anti-mouse CD16/CD32 antibody and stained with Fixable Viability Dye eFluor 780, Alexa488 anti-mouse CD90.2 antibody (1:200 dilution; clone:30-H12; BioLegend), PE anti-mouse TCR β chain antibody (1:200 dilution; clone: H57-597; BioLegend), BV605 anti-mouse CD8a antibody (1:200 dilution; clone: 53-6.7; BioLegend), and PE anti-mouse CD4 antibody (1:200 dilution; clone: RM4-5; BioLegend) in PBS with 2% FBS, 1 mM EDTA, and 0.05% sodium azide in dark. Cells were enumerated using flow cytometry by adding AccuCheck Counting Beads (Thermo Fisher Scientific) to the samples.

Supplemental Figure 5: NALT lymphocytes, nasal passage lymphocytes, and splenocytes ($1\text{-}3 \times 10^6$ cells) derived from IAV-mice immunized with Spike-HA were collected for cytokine production evaluation using flow cytometry. Fourteen days after the final immunization, cells were treated with Spike (50 μg/mL) in RPMI1640 with 10% FBS, 1% penicillin-streptomycin, and 50 mM 2-mercaptoethanol and incubated for 19 h at 37°C. After incubation, 1:500 diluted protein transport inhibitor cocktails (Thermo Fisher Scientific) were kept for 5 h at 37°C in 96 well plates. Following this, cells were blocked with anti-mouse CD16/CD32 antibody and stained with Fixable Viability Dye eFluor 780, Alexa647 anti-mouse CD45 antibody, PE anti-mouse CD3ε antibody (1:200 dilution; clone: 145-2C11; BioLegend), FITC anti-mouse CD4 antibody (1:200 dilution; clone: GK1.5; BioLegend), BV605 anti-mouse CD8a antibody, BV510 anti-mouse CD44 antibody (1:200 dilution; clone: IM7; BioLegend) in PBS with 2% FBS, 1 mM EDTA, and 0.05% sodium azide for 30 min at 4°C in dark, followed by intracellular BV421 anti-IFN-γ antibody (1:200 dilution; clone: XMG1.2; BioLegend) and PE/Cy7 anti-IL-13 antibody (1:200 dilution; clone: eBio13A; Thermo Fisher Scientific) staining using a BD Cytofix/Cytoperm™ Fixation/ Permeablization solution Kit in accordance with the manufacturer's instructions.

Supplemental Methods

Preparation of mRNA vaccine (Figure 7 and Supplemental Figure 12)

For transcription of mRNA expressing the Spike of SARS-CoV-2, template pDNA encoding Spike was prepared by PCR. The linearized pDNA was transcribed into mRNA by MEGAscript™ T7 transcription kit (Thermo Fisher Scientific) according to the manufacturer's instructions. N1-methylpseudouridine was incorporated into the mRNA by replacing uridine. The 5′ cap was added according to the protocol of ScriptCap Cap 1 Capping System (C-SCCS1710, Madison, WI, USA). The 3′ poly(A) tail was added according to the protocol of poly(A) Tailing Kit (AM1350, Thermo Fisher Scientific). We used a lipid mixture containing SM-102 (Cayman Chemical Company, Ann Arbor, MI, USA), DMG-PEG2000 (NOF Corporation, Tokyo, Japan), DSPC (NOF Corporation), and cholesterol (Sigma-Aldrich) in an ethanol solution (SM-102/DSPC/cholesterol/DMG-PEG2000 = 50/10/38.5/1.5). The LNP-mRNA was produced by mixing the mRNA dissolved in acetic acid/NaOH buffer (pH 5.0) and the lipid mixture in ethanol at an N/P ratio of 5.5 using NanoAssemblr® instrument (Precision Nanosystems, Vancouver, Canada) at a flow rate ratio of 3:1. The external solution of LNP-mRNA was replaced by Dulbecco's phosphate buffered saline (D-PBS) (Nacalai Tesque) by ultrafiltration using Amicon Ultra-4-100K centrifugal units. The size and zeta potential of the LNP-mRNA were measured by Zetasizer Nano ZS (Malvern Instruments, Malvern, UK). Encapsulation efficiency of the mRNA was measured by Ribogreen™ reagent (Invitrogen, San Diego, CA, USA).

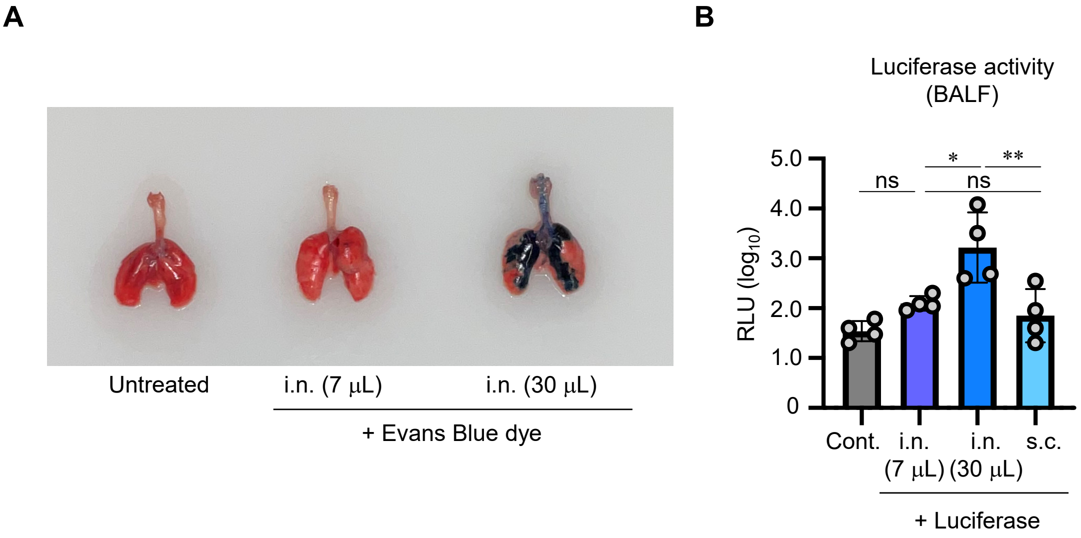

Supplemental Figure 1. Intranasal administration of a small fluid volume can limit antigen delivery to the upper respiratory tract of mice.

(A) Representative images of airways and lungs from mice that were either untreated or treated with Evans Blue dye at volumes of 7 μL or 30 μL 24 h after administration. (B) Mice were intranasally administrated 1 × 10^6 units of luciferase protein at a volume of 7 μL or 30 μL. BALF was obtained two h after administration. Measurements of luciferase activity in BALF of untreated or luciferase protein-treated mice were taken. Data are represented as means ± SD. (B) n = 4. Each experiment was performed more than twice. *$P < 0.05$; **$P < 0.01$ as indicated by Tukey's multiple comparisons test. ns, not statistically significant.

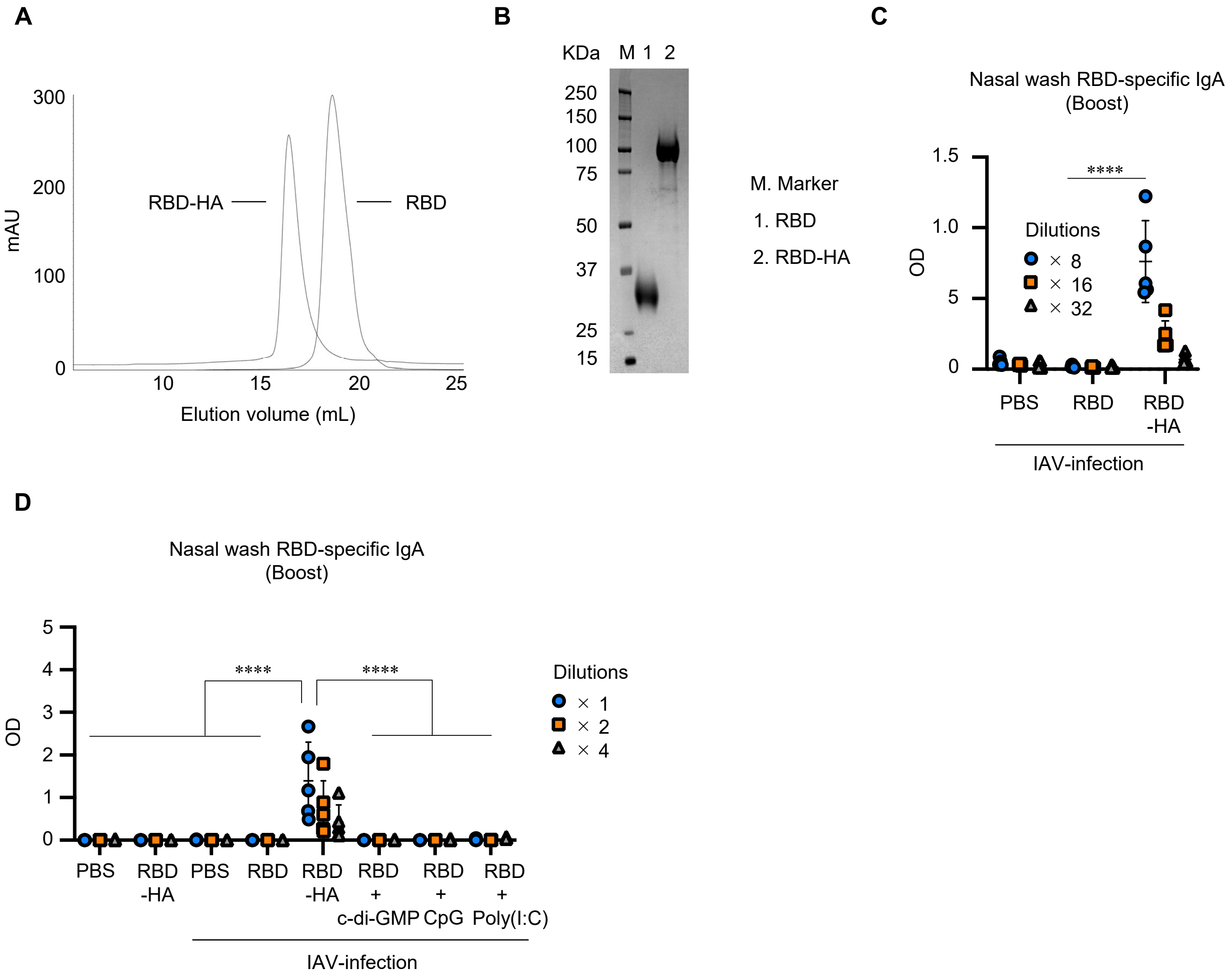

Supplemental Figure 2. Construction and characterization of recombinant RBD-HA.

(A) Recombinant RBD and RBD-HA generated in Expi293F cells were purified by size-exclusion chromatography after metal ion chromatography. (B) Purified recombinant RBD and RBD-HA were analyzed using SDS-PAGE followed by staining with Coomassie Brilliant Blue. M, marker; lane 1, RBD; lane 2, RBD-HA. (C) BALB/c mice were infected intranasally with IAV, followed by intranasal immunization with RBD or RBD-HA at 30 and 51 days after IAV infection. The RBD-specific IgA levels in nasal washes after booster immunizations were evaluated using an ELISA. (D) C57BL/6 mice were infected intranasally with IAV, followed by intranasal immunization with either RBD-HA, RBD plus c-di-GMP, RBD plus CpG ODN, or RBD plus poly(I:C) at 30 and 51 days after IAV infection. The RBD-specific IgA levels in nasal washes after booster immunizations were evaluated using an ELISA. (C and D) Data are represented as mean ± SD. (C and D) n = 5. Each experiment was performed more than twice. ****$P < 0.0001$ as indicated by Tukey' multiple comparisons test.

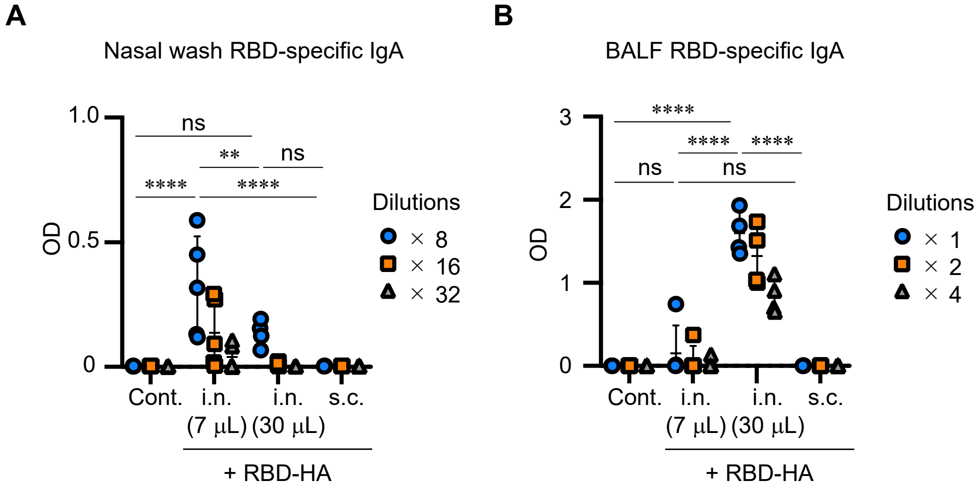

Supplemental Figure 3. Evaluation of antibody production in different fluid volumes.

(A and B) IAV-mice were immunized with RBD-HA either subcutaneously or intranasally at volumes of 7 μL or 30 μL. The RBD-specific (A) nasal wash IgA and (B) BALF IgA levels were evaluated using an ELISA after booster immunizations. (A and B) n = 4-5. (A and B) Data are represented as means ± SD. Each experiment was performed more than twice. ***P* < 0.01; *****P* < 0.0001 as indicated by Tukey's multiple comparisons test. ns, not statistically significant.

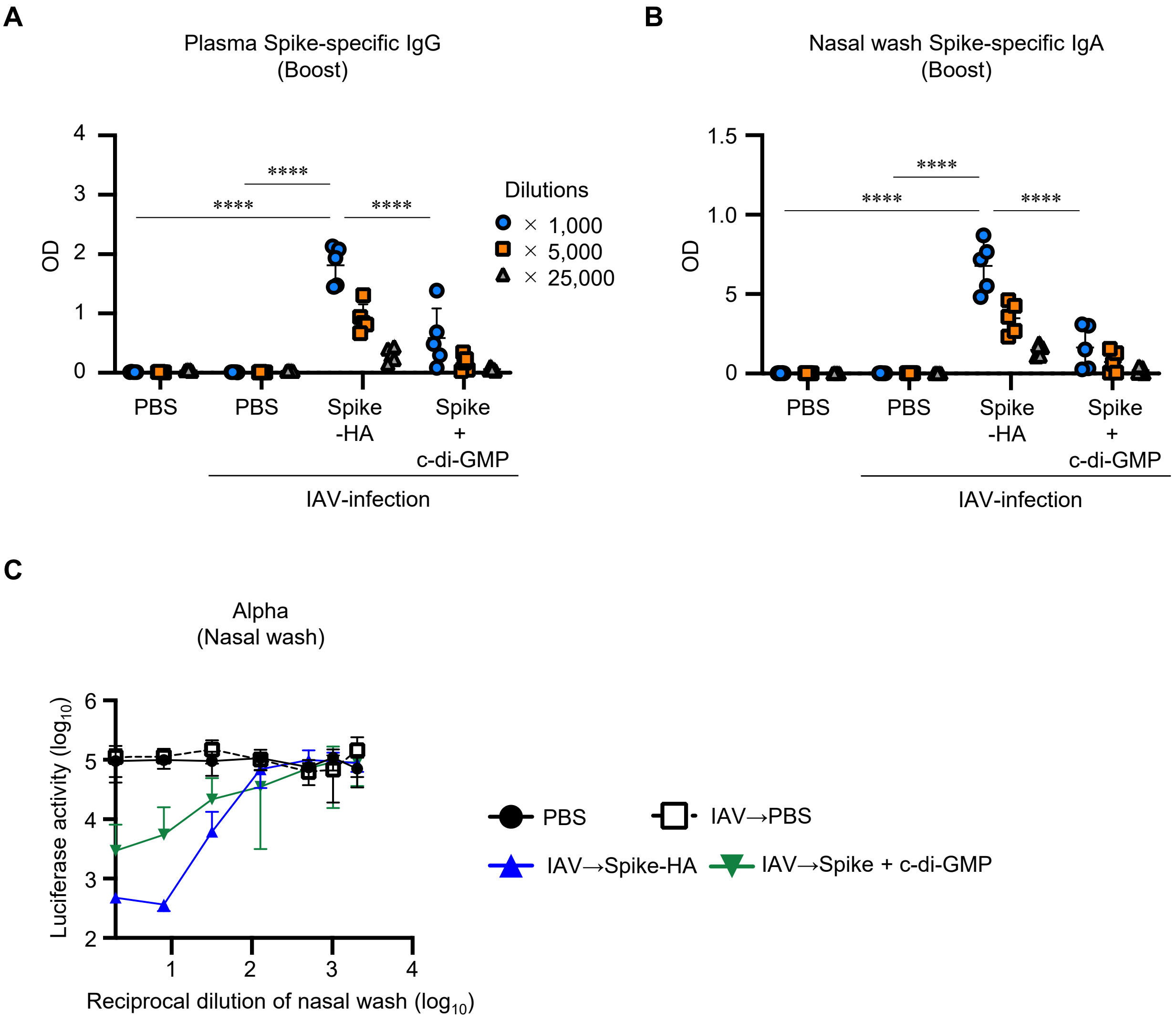

Supplemental Figure 4. Intranasal immunization with Spike-HA can induce Spike-specific antibodies. (A and B) IAV-mice were immunized intranasally with Spike-HA or Spike plus c-di-GMP at 30 and 51 days after IAV infection. The levels of Spike-specific (A) IgG in plasma and (B) IgA in nasal washes were evaluated using an ELISA after booster immunizations. (C) Neutralization measurements against vesicular stomatitis virus-based pseudotyped viruses displaying the Alpha Spike of SARS-CoV-2 in nasal washes. (A-C) Data are represented as mean ± SD. n = 5. Each experiment was performed more than twice. ****$P < 0.0001$ as indicated by Tukey's multiple comparisons test.

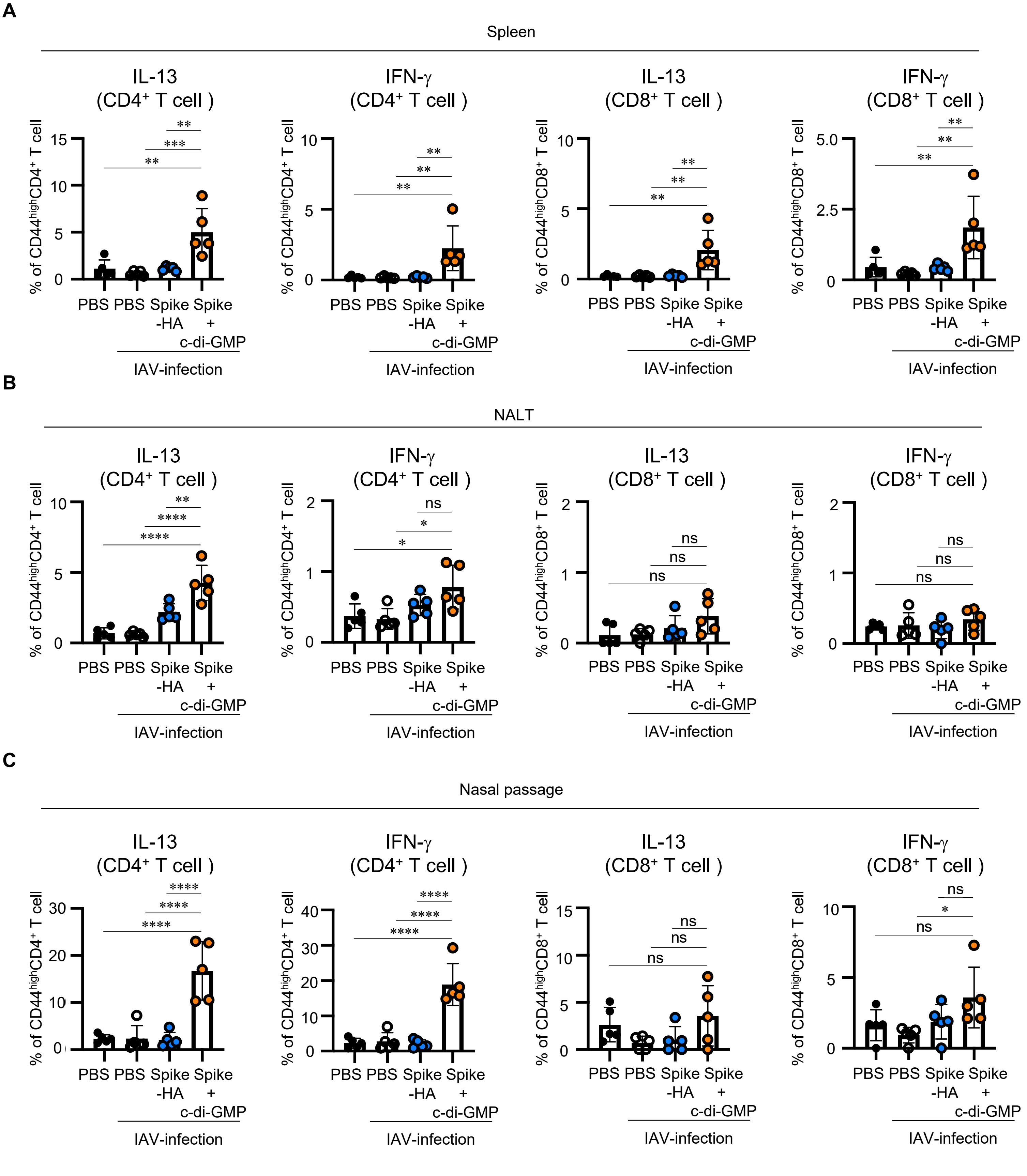

Supplemental Figure 5. Intranasal immunization with Spike-HA is inefficient in inducing T cell responses.

(A-C) Spike-specific $CD4^+$ and $CD8^+$ T cell responses in (A) spleen, (B) NALT, and (C) nasal passage 14 days after booster immunization were evaluated by flow cytometry. (A-C) Data are represented as mean ± SD. n = 5. Each experiment was performed more than twice. *$P < 0.05$; **$P < 0.01$; ***$P < 0.001$; ****$P < 0.0001$ as indicated by Tukey' multiple comparisons test. ns, not statistically significant.

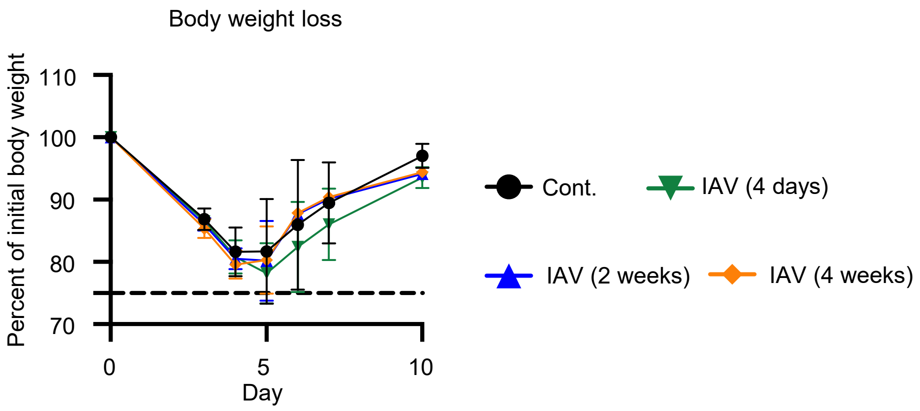

Supplemental Figure 6. IAV infection did not protect against SARS-CoV-2 challenge.

Mice were infected with SARS-CoV-2 at different time points following IAV infection (4 days, 2 weeks, or 4 weeks). After the SARS-CoV-2 challenge, the percentage changes in body weight were monitored. Data are represented as mean ± SD. n = 5. The experiment was performed twice.

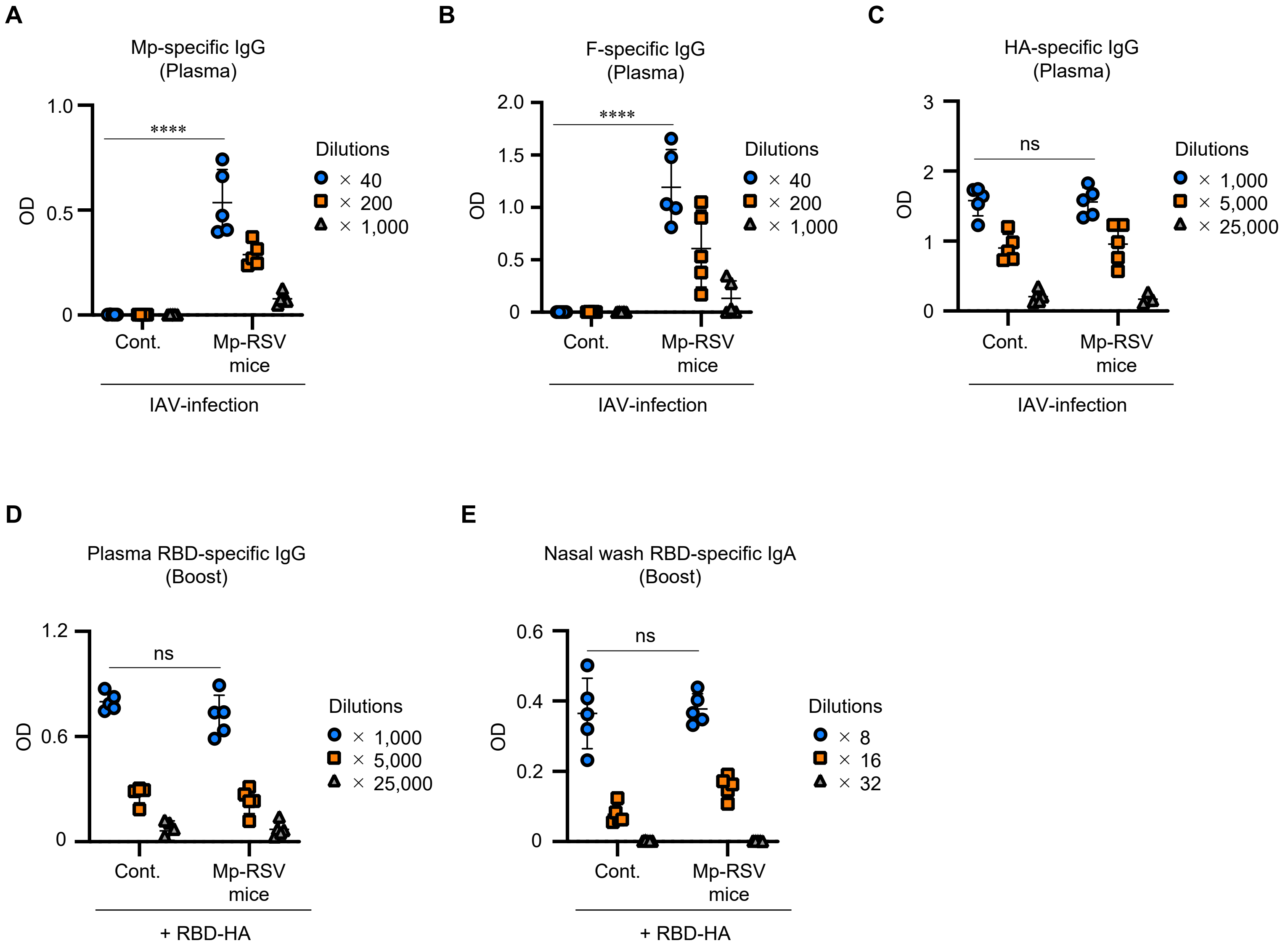

Supplemental Figure 7. Induction of immunity by RBD-HA is not affected by previous infections with other pathogens.

(A-E) Mice were sequentially infected with Mp and respiratory syncytial virus (Mp-RSV mice), then IAV infection, followed by intranasal immunization with RBD-HA at 30 and 51 days after IAV infection. The levels of (A) Mp-, (B) F-, the major membrane protein of respiratory syncytial virus, and (C) HA-specific IgG in plasma were evaluated using an ELISA 28 days after IAV infection. The RBD-specific (D) plasma IgG levels and (E) nasal wash IgA levels were evaluated using after booster immunizations. (A-E) n = 5. Data are represented as means ± SD. Each experiment was performed more than twice. ****$P < 0.0001$ as indicated by Tukey's multiple comparisons test. ns, not statistically significant.

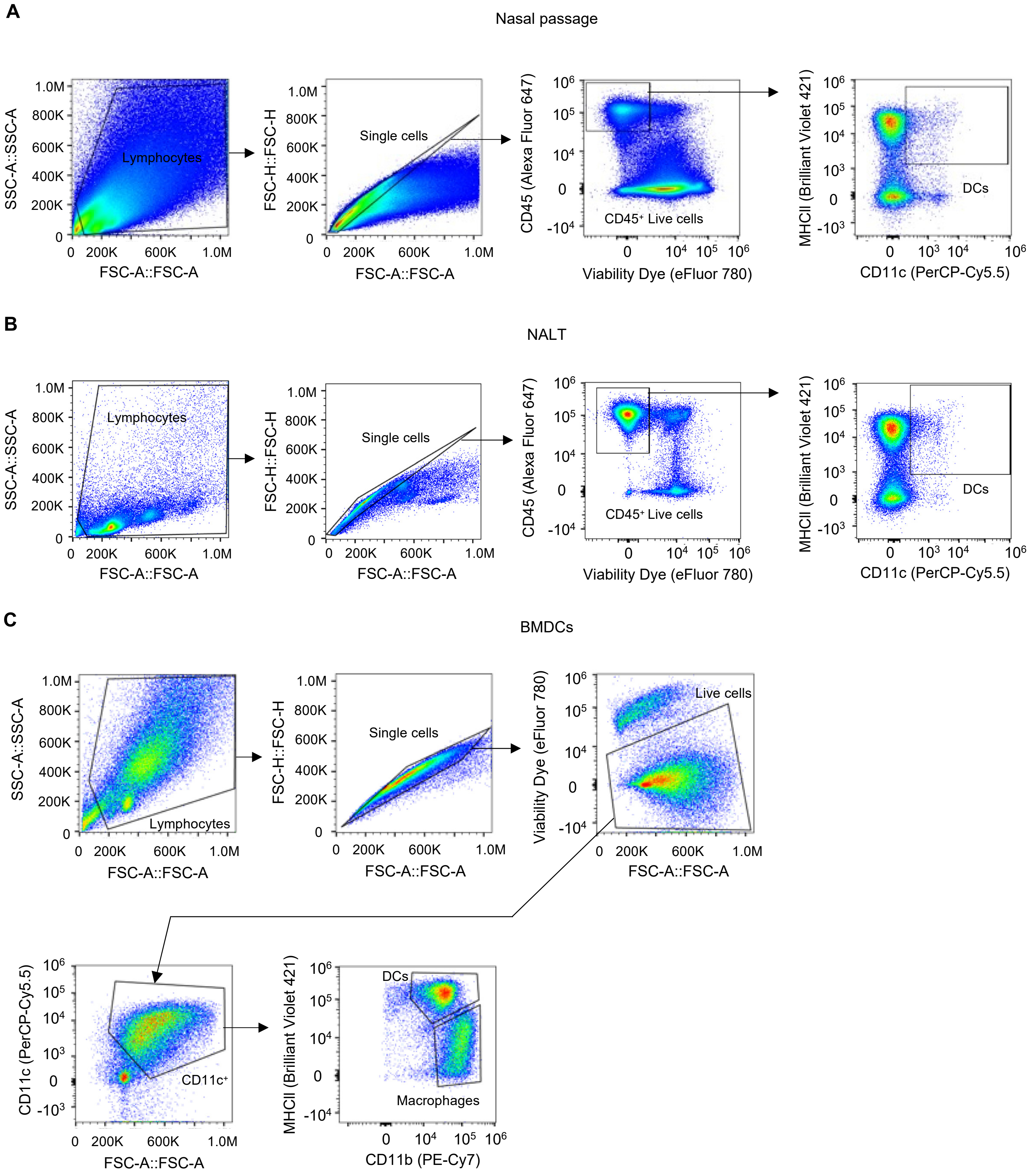

Supplemental Figure 8. Gating strategies for analysis of DCs responses in nasal passage and NALT. (A) Gating strategy to identify uptake of EGFP by DCs in nasal passage. (B) Gating strategy for analysis of CD86, a co-stimulatory molecule, expression on DCs in NALT. (C) Gating strategy to identify BMDCs in bone marrow-derived DC with GM-CSF.

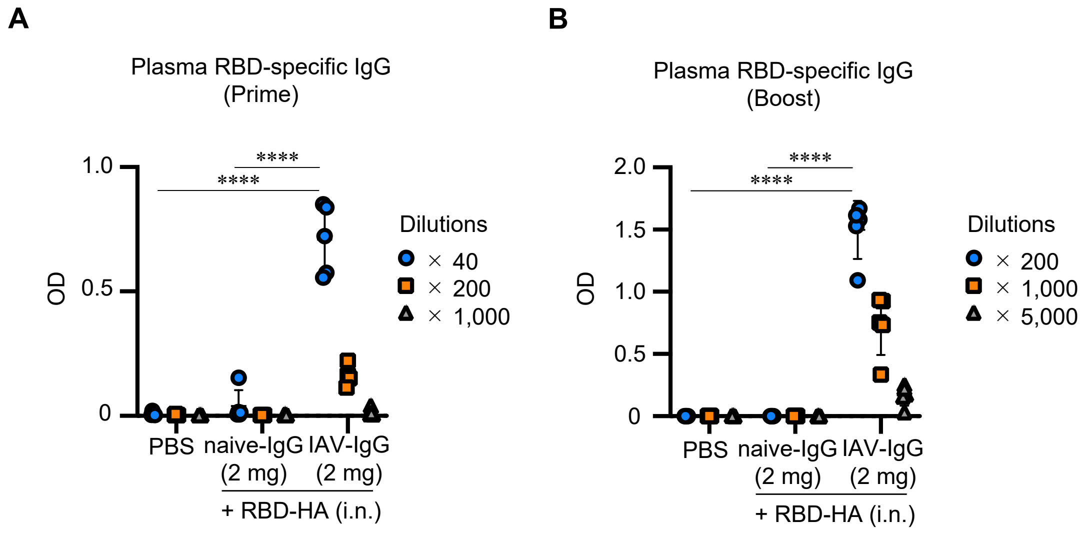

Supplemental Figure 9. HA-specific purified IgG contribute to the immune responses induced by intranasal vaccination with RBD-HA.

(A and B) BALB/c mice received 2 mg of purified naive-IgG or IAV-IgG intraperitoneally and were immunized intranasally after 24 h with RBD-HA. The RBD-specific IgG levels were evaluated using an ELISA (A) plasma IgG levels after primary immunization and (B) plasma IgG levels after booster immunization. (A and B) Data are represented as mean ± SD. n = 5. Each experiment was performed more than twice. ****$P < 0.0001$ as indicated by Tukey's multiple comparisons test.

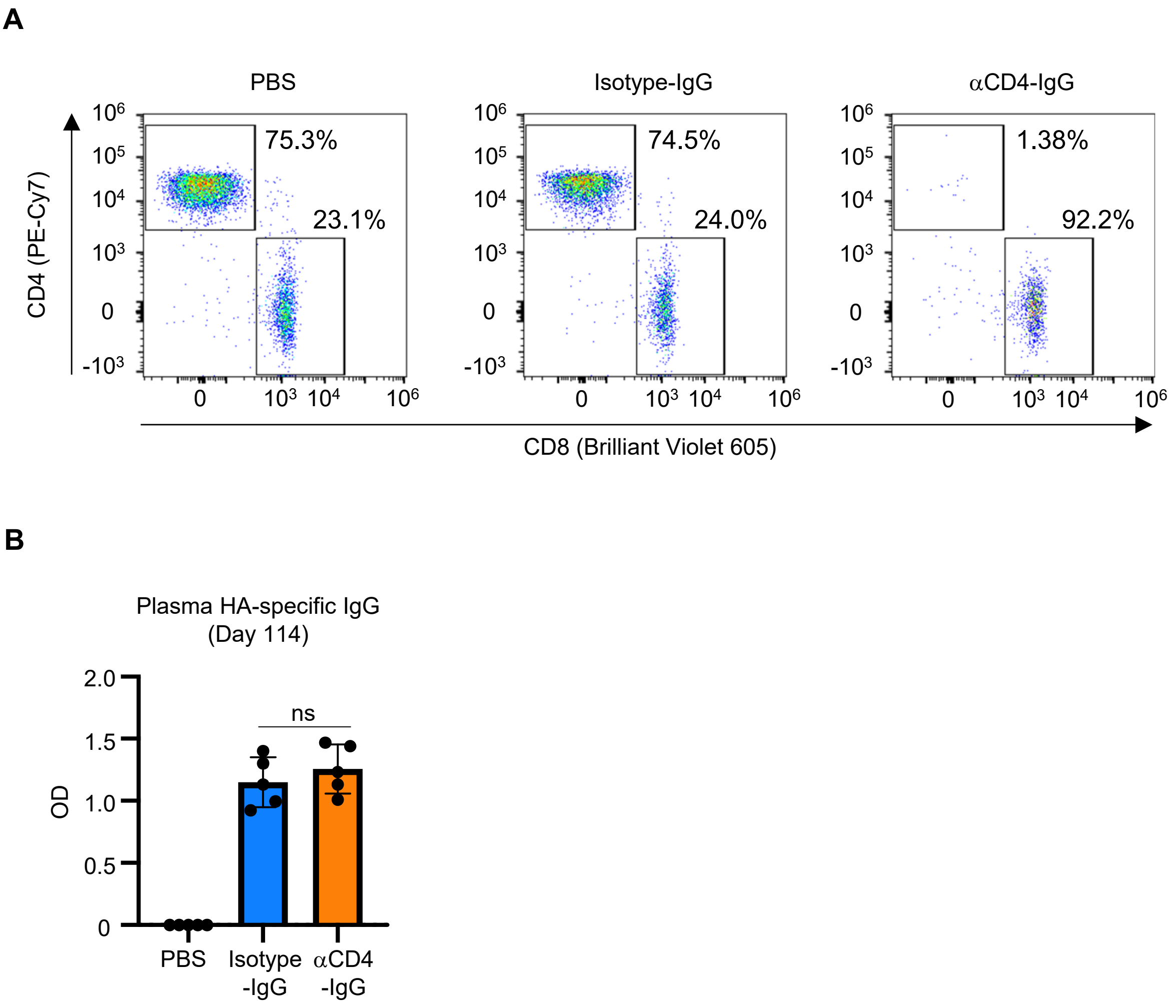

Supplemental Figure 10. T cell depletion.

(A and B) To deplete HA-specific pre-existing CD4$^+$ T cells, IAV-mice were injected intraperitoneally with 200 μg anti-CD4 antibody (GK1.5) or isotype antibody at days 30 and 32, respectively, after IAV infection. (A) Representative flow plots of gate on live CD45$^+$ CD90.2$^+$ TCR-β$^+$ cells are shown two days after injection with depletion antibody. (B) The plasma HA-specific IgG levels two days prior to immunization with RBD-HA. We used 200-fold dilutions of plasma samples. (B) Data are represented as mean ± SD. n = 5. Each experiment was performed more than twice. P-values were determined by Tukey's multiple comparisons test. ns, not statistically significant.

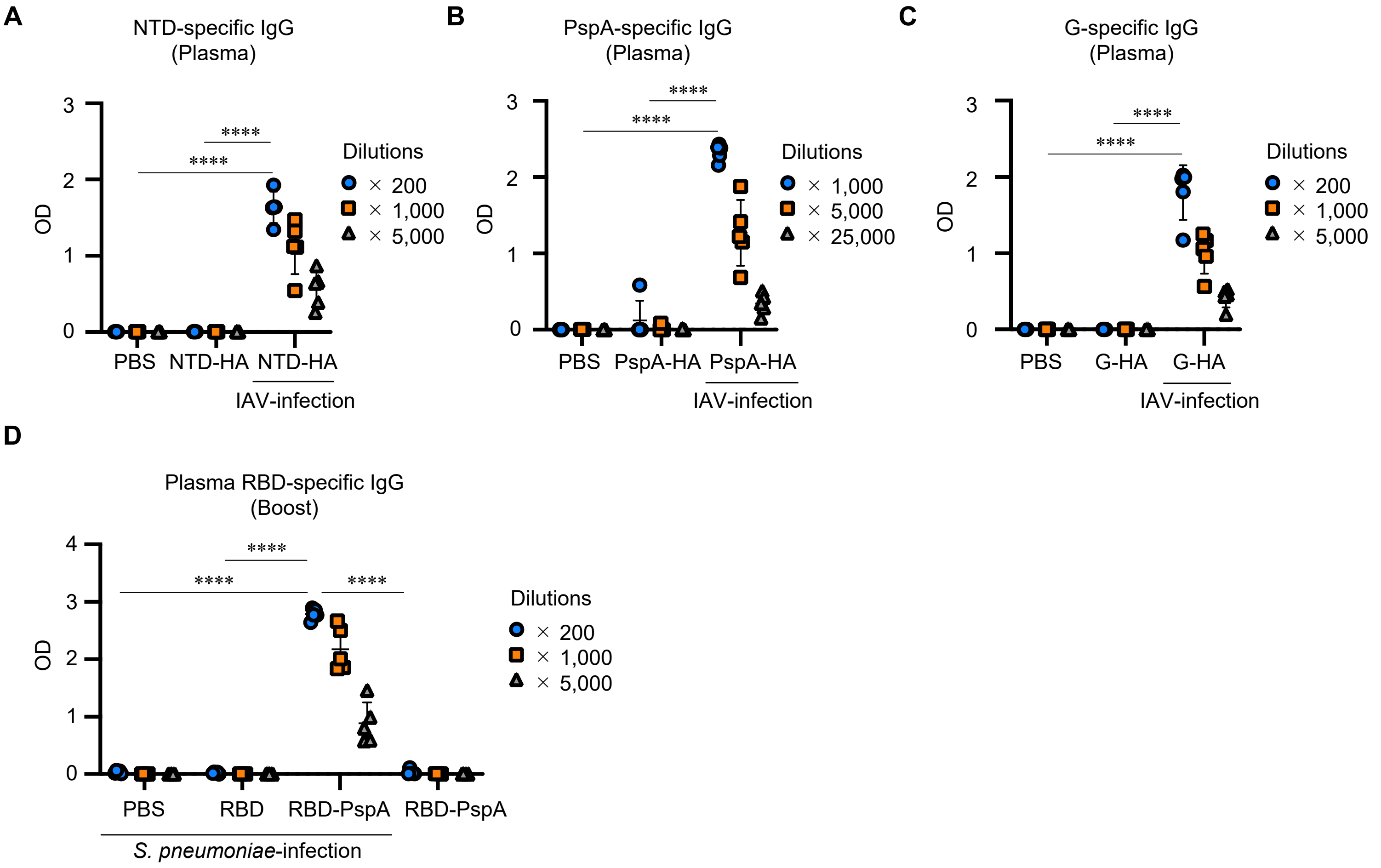

Supplemental Figure 11. Intranasal subunit vaccine platform that utilizes pre-existing immunity is highly versatile.

(A-C) IAV-mice were immunized intranasally with (A) NTD-HA, (B) PspA-HA, or (C) G-HA at 30 and 51 days after IAV infection. (A-C) The levels of (A) NTD-, (B) PspA-, and (C) G-specific IgG in plasma after booster immunization were evaluated using an ELISA. (D) BALB/c mice were infected intranasally with *S. pneumoniae*, followed by intranasal immunization with RBD-PspA or RBD without adjuvant at 30 and 51 days after *S. pneumoniae* infection. The RBD-specific plasma IgG levels were evaluated using an ELISA. (A-D) Data are represented as mean ± SD. n = 5. Each experiment was performed more than twice. ****$P <$ 0.0001 as indicated by Tukey's multiple comparisons test.

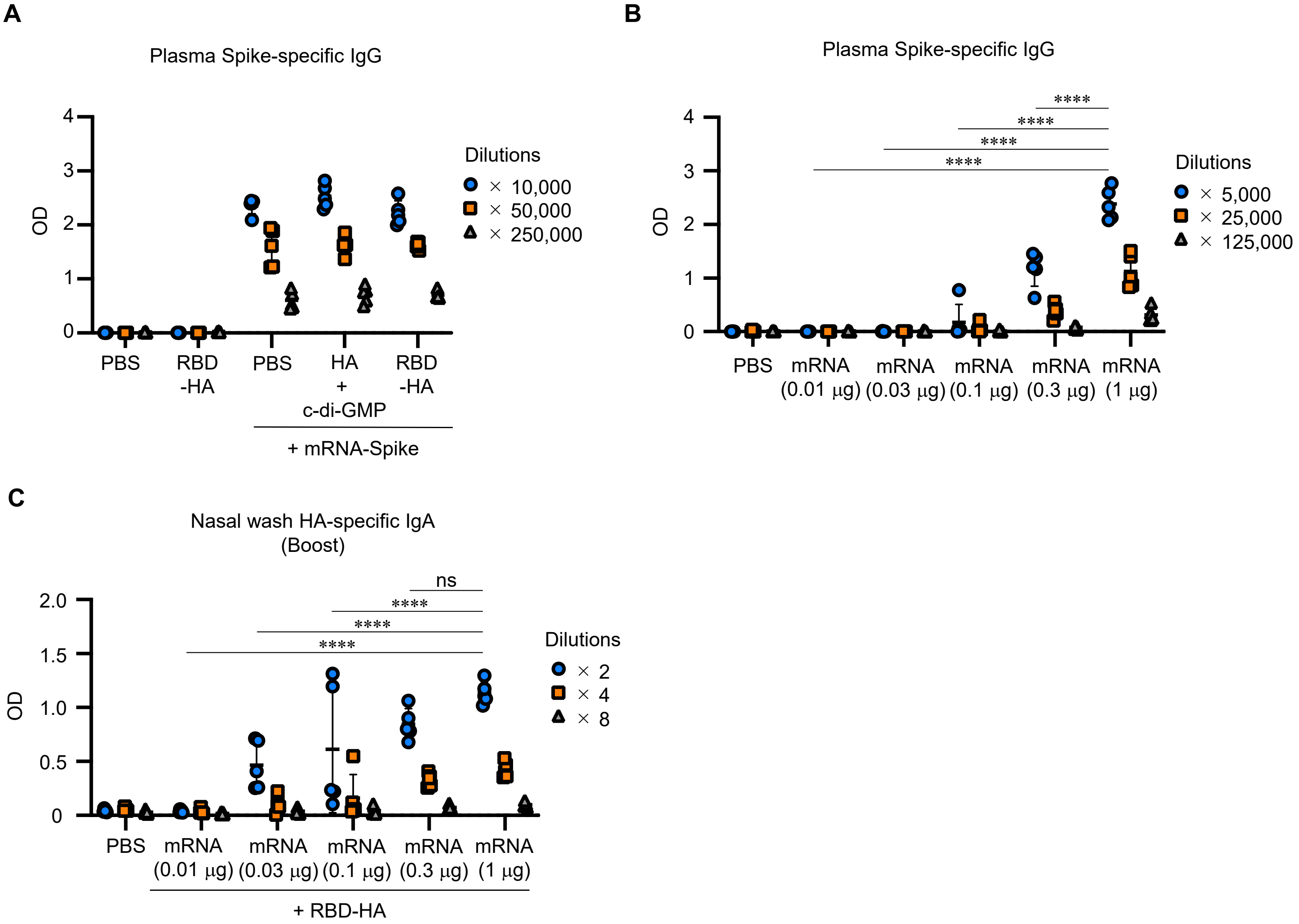

Supplemental Figure 12. Antibody production induced by RBD-HA varies depending on the level of pre-existing immunity.

(A) BALB/c mice were subcutaneously immunized with 1 μg of mRNA vaccine encoding SARS-CoV-2 Spike twice. The plasma Spike-specific IgG levels seven days after booster immunizations were evaluated using an ELISA. (B and C) BALB/c mice were immunized subcutaneously twice with 0.01, 0.03, 0.1, 0.3, or 1 μg of mRNA vaccine encoding SARS-CoV-2 Spike, followed by intranasal immunization with RBD-HA at 30 and 51 days after mRNA vaccine. (B) The plasma Spike-specific IgG levels seven days after booster immunizations were evaluated using an ELISA. (C) The HA-specific nasal wash IgA levels 14 days after booster immunizations with RBD-HA were evaluated using an ELISA. (A-C) Data are represented as mean ± SD. n = 5. Each experiment was performed more than twice. ****$P < 0.0001$ as indicated by Tukey's multiple comparisons test. ns, not statistically significant.

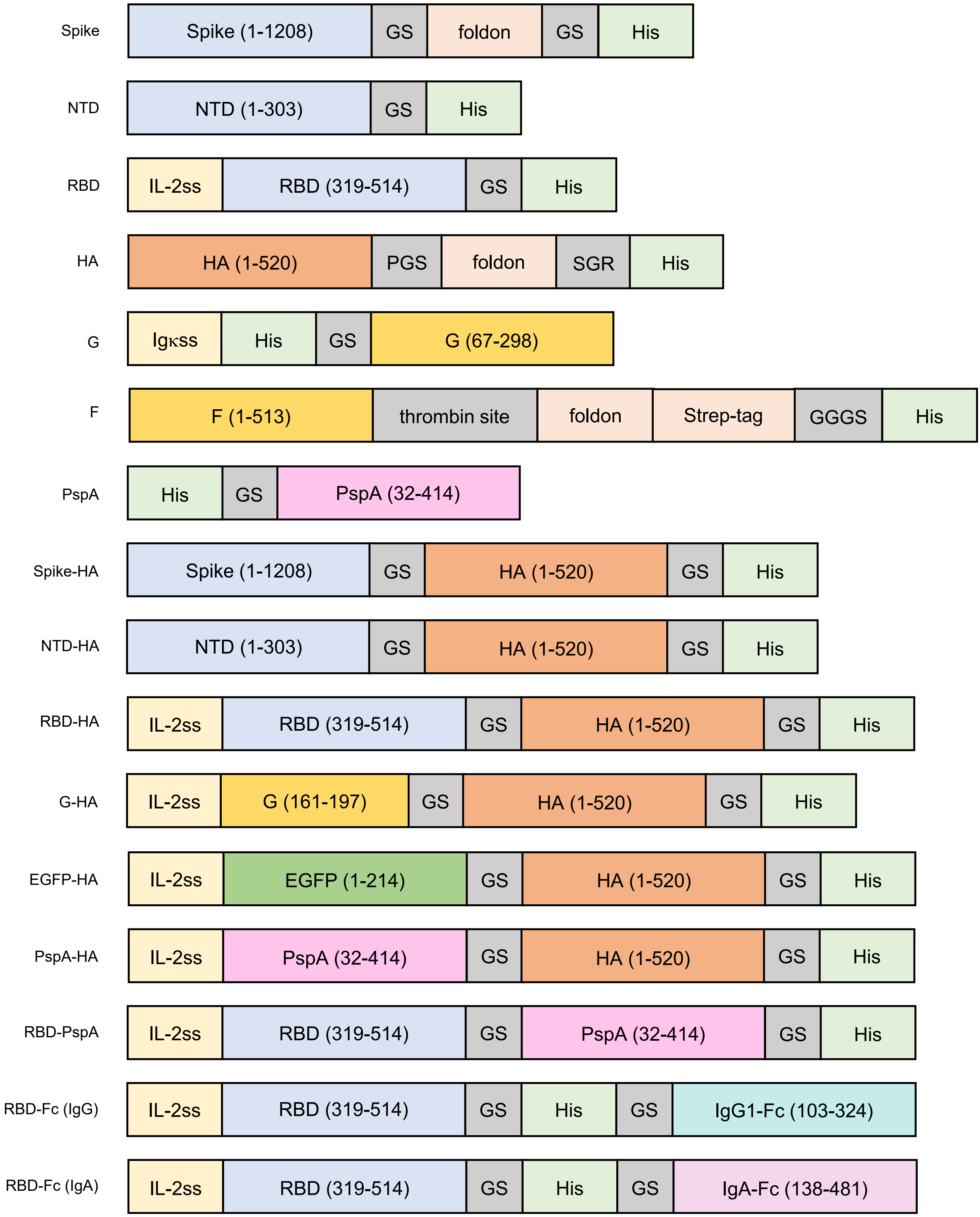

Supplemental Figure 13. Construction of recombinant proteins.

Schematic representation of protein structures.

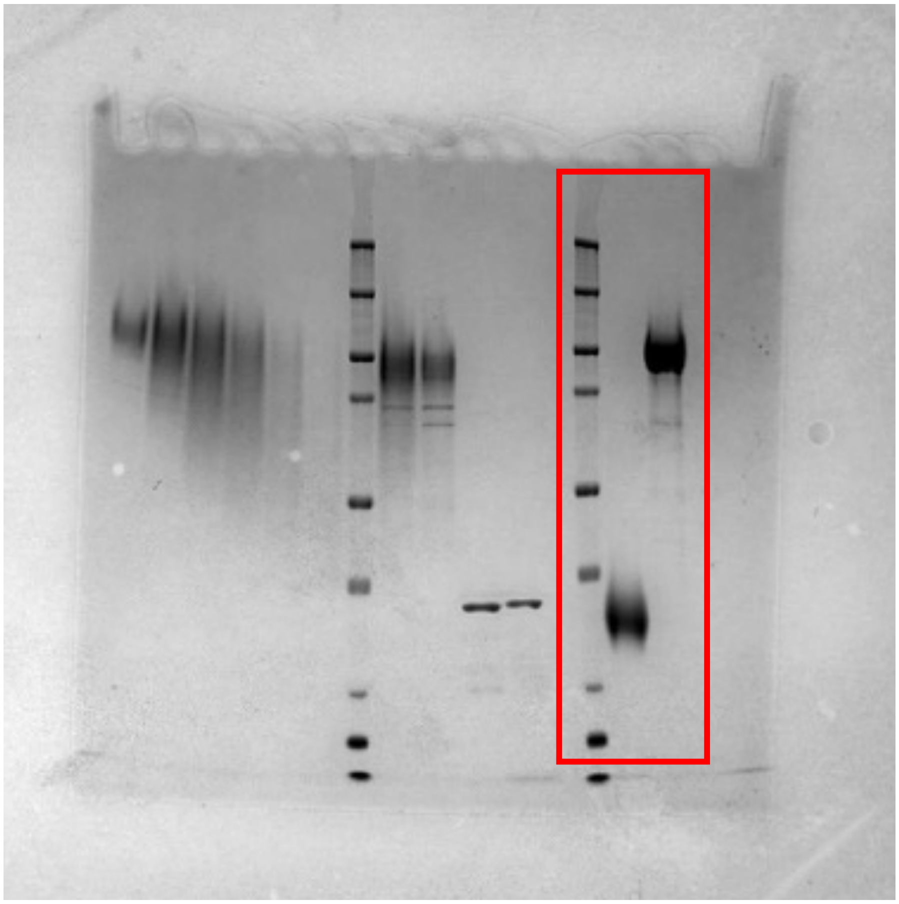

Full unedited gel for Supplemental Figure 2B
